# Castor RcnsLTPC Confers Salt Tolerance in Yeast and Tobacco with Synergistic Enhancement by ZnO-NPs Priming

**DOI:** 10.3390/plants15121827

**Published:** 2026-06-12

**Authors:** Peilin Han, Bing Gao, Yingxin Han, Yueming Li, Jinghong Wang, Jixiang Lin

**Affiliations:** College of Landscape Architecture, Northeast Forestry University, Harbin 150040, China; han-peilin@nefu.edu.cn (P.H.);

**Keywords:** castor (*Ricinus communis* L.), *RcnsLTPC*, nonspecific lipid transfer protein, ZnO-NPs priming, salt tolerance

## Abstract

Soil salinity severely restricts castor (*Ricinus communis* L.) seed germination, yet the molecular basis of this trait remains poorly understood. Here, we identified and functionally characterized *RcnsLTPC*, a nonspecific lipid transfer protein gene strongly induced by salt stress, which encodes a plasma membrane-localized nsLTP1 protein. Promoter analyses indicated that RcnsLTPC is responsive to stress-, hormone-, and light-related signals, supporting its potential role in environmental adaptation. Heterologous expression in *Saccharomyces cerevisiae* and overexpression in *Nicotiana tabacum* consistently demonstrated that *RcnsLTPC* acts as a positive regulator of salt tolerance, improving germination, root development, biomass accumulation, antioxidant capacity, and ion homeostasis under NaCl stress. Notably, ZnO-NPs priming further amplified the protective effects of *RcnsLTPC*, suggesting a synergistic interaction between nanopriming and gene-mediated stress adaptation. Collectively, these findings establish *RcnsLTPC* as a key regulator of salt tolerance in castor and provide a conceptual basis for combining nanotechnology with genetic enhancement to improve crop performance on saline soils.

## 1. Introduction

Soil salinization is one of the most severe abiotic constraints on global agricultural productivity, affecting approximately 20% of arable land and nearly 50% of irrigated farmland worldwide, with ongoing expansion driven by climate change and inappropriate irrigation [1,2]. Salt stress impairs plant growth through osmotic imbalance and ionic toxicity, causing excessive reactive oxygen species (ROS) accumulation and crop yield losses of up to 50% [3,4]. Among all developmental stages, seed germination is particularly vulnerable to salinity, and failure at this stage directly leads to poor field emergence and reduced yield potential [5]. Therefore, improving salt tolerance during germination is of great importance for maintaining crop productivity on saline and marginal lands.

Castor (*Ricinus communis* L.), a member of the Euphorbiaceae family, ranks among the top ten oilseed crops worldwide, with seeds containing 40–60% oil predominantly composed of ricinoleic acid (~90% of total fatty acids), conferring significant industrial value [6,7]. Castor exhibits rapid growth, an extensive root system, and notable tolerance to drought and nutrient-poor soils, making it a strategic crop for reclaiming saline-alkaline and marginal lands. However, castor is moderately sensitive to salinity, with NaCl concentrations exceeding 100 mM markedly inhibiting seed germination and reducing seed biomass by over 50%, thereby severely limiting its large-scale cultivation in saline regions [8,9]. The high content of unsaturated fatty acids, particularly ricinoleic acid, in castor seeds may provide an inherent advantage for maintaining membrane fluidity and mitigating osmotic stress. Nevertheless, the molecular mechanisms underlying salt tolerance during germination remain largely unexplored, representing a critical knowledge gap that hinders the breeding of salt-tolerant castor cultivars [10].

Seed priming is a pre-sowing treatment widely recognized for enhancing germination quality and stress resilience by pre-activating metabolic and transcriptional programs within the seed [11]. Among emerging priming strategies, zinc oxide nanoparticle (ZnO-NPs)-based priming has attracted considerable attention due to the ability of nanoparticles (1–100 nm) to penetrate the seed coat and trigger pronounced physiological responses at low dosages [12]. ZnO-NPs have been demonstrated to alleviate salinity-induced germination inhibition in multiple crop species, with mechanisms primarily attributed to sustained Zn^2+^ release, which activates antioxidant enzyme systems and stabilizes plasma membrane structure [13,14]. However, whether ZnO-NPs priming modulates lipid metabolism-related gene expression and synergizes with endogenous salt tolerance genes remains unclear, particularly in castor.

Nonspecific lipid transfer proteins (nsLTPs) constitute a widely distributed class of multifunctional small basic proteins in plants, characterized by a conserved eight-cysteine motif that forms a hydrophobic cavity capable of binding and transporting diverse polar lipid molecules in vitro [15,16]. The nsLTP family is encoded by multiple genes, with over 50 members identified in model and major crops such as *Arabidopsis thaliana*, rice, and maize, and can be phylogenetically classified into several subtypes, including LTP1 and LTP2 [17]. The biological functions of nsLTPs extend far beyond conventional lipid transport, encompassing pathogen defense, cuticular wax biosynthesis, mobilization of storage lipids during seed germination, and responses to abiotic stress [17]. Notably, overexpression of specific nsLTP genes markedly enhances salt tolerance in transgenic plants; for instance, wheat overexpressing *TdLTP*4 and soybean lines overexpressing *GmLtpI.3* display improved survival under NaCl and drought stress, respectively [15,18]. In castor seeds, nsLTP proteins were previously localized to glyoxysomes, suggesting a potential role in fatty acid β-oxidation during germination [16]. Nevertheless, the identification and functional characterization of castor nsLTP family members remain extremely limited, and their regulatory roles under salt stress have yet to be systematically elucidated.

In our previous study, an integrative lipidomic and transcriptomic analysis of castor seeds primed with ZnO-NPs showed that, under 100 mM NaCl stress, ZnO-NPs priming markedly upregulated the expression of the castor nsLTP gene *RcnsLTPC* (LOC8287900). These transcriptional changes were accompanied by coordinated alterations in phosphatidylcholine (PC), phosphatidic acid (PA), and phosphatidylethanolamine (PE), suggesting that *RcnsLTPC* may link nanoparticle-mediated priming to membrane lipid remodeling [19]. However, the protein structural features, subcellular localization, and independent functional role of *RcnsLTPC* under salt stress remain unverified through direct genetic evidence, and the precise molecular mechanisms by which it regulates plant salt tolerance require systematic elucidation.

To address these knowledge gaps, the present study used germinating castor seeds as the experimental material to systematically clone and characterize the *RcnsLTPC* gene, elucidate the structural features and subcellular localization of its encoded protein, and perform multi-level functional validation of its salt tolerance using a heterologous yeast expression system and transgenic tobacco models. Building on this foundation, we further evaluated the synergistic effects of ZnO-NPs priming and *RcnsLTPC* overexpression on key salt tolerance traits, including germination rate, biomass accumulation, ROS scavenging, ion homeostasis, and root architecture. By integrating physiological, cellular, and molecular analyses, we aimed to clarify the mechanisms underlying *RcnsLTPC*-mediated salt stress tolerance. This study not only provides new experimental evidence for the functional diversity of the castor nsLTP family but also provides a scientific basis for the practical application of a “nanopriming + functional gene enhancement” strategy to improve saline-alkaline soils.

## 2. Results

### 2.1. Cloning and Sequence Verification of RcnsLTPC

**Figure 1:**
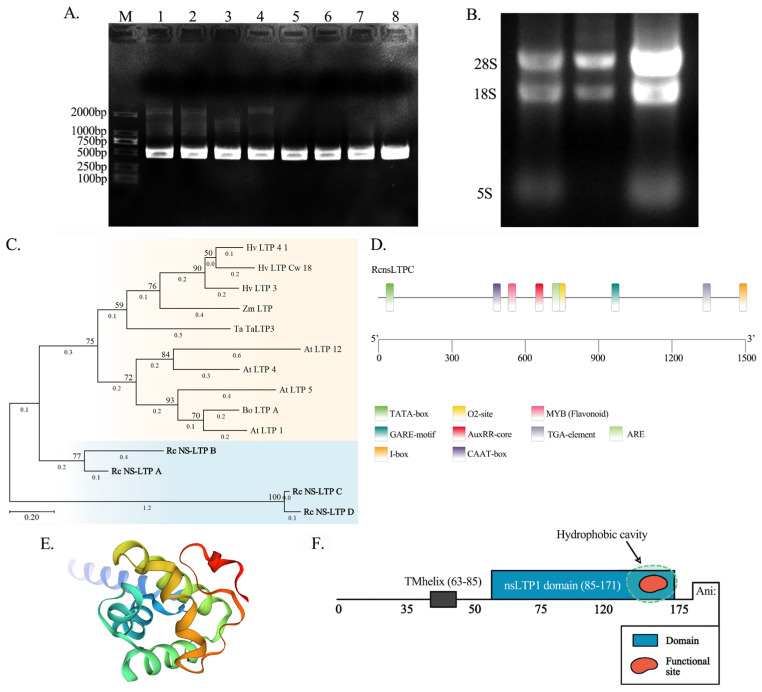
Electrophoretic analysis of PCR amplification products of the *RcnsLTPC* gene coding region (**A**) and total RNA quality control of castor seeds (**B**), where lane M is the DL2000 DNA marker (band sizes in bp) and lanes 1–8 are target fragment amplification products. Shown are also the phylogenetic analysis based on amino acid sequences (**C**), with nodal values indicating bootstrap support rates (1000 replicates) and background color denoting different clades; the distribution of *cis*-acting elements in the *RcnsLTP*C gene promoter region (**D**), with colored rectangles representing distinct element types; the tertiary structure spatial conformation model of the RcnsLTPC protein (**E**); and the schematic diagram of the linear structural domains of the RcnsLTPC protein (**F**).

The full-length coding sequence of *RcnsLTPC* was successfully amplified by PCR using gene-specific primers and cDNA synthesized from total RNA extracted from germinating castor bean seeds. Agarose gel electrophoresis revealed a single, discrete band of approximately 500 bp (Figure 1A), consistent with the predicted ORF size based on transcriptome assembly. The amplicon was cloned into the pEASY^®^-Blunt vector and confirmed by Sanger sequencing, establishing that the *RcnsLTPC* CDS is 528 bp and encodes a 175-amino-acid protein. NCBI BLAST (version 2.16.0) analysis mapped this sequence to a putative nsLTP gene annotated in the castor reference genome (RefSeq accession: NC_063259), identifying *RcnsLTPC* as a novel member of the castor nsLTP gene family. Total RNA used for reverse transcription displayed intact 28S/18S ribosomal RNA bands by gel electrophoresis, with A260/280 ratios of 1.8–2.0 (Figure 1B), confirming sufficient RNA integrity and purity for downstream analyses.

### 2.2. Phylogenetic Analysis and Promoter Cis-Element Identification

#### 2.2.1. Phylogenetic Relationships

A neighbor-joining phylogenetic tree constructed from full-length amino acid sequences resolved plant nsLTP proteins into two major clades (Figure 1C). *RcnsLTPC* clustered with the endogenous castor bean paralogs RcnsLTPA, RcnsLTPB, and RcnsLTPD into a monophyletic group supported by 100% bootstrap confidence, suggesting that these proteins arose through a recent tandem duplication event within the castor bean genome. Notably, this castor bean-specific clade showed relatively close phylogenetic affinity to LTP proteins from monocot species, including barley and maize, indicating that RcnsLTPC retains a degree of sequence conservation across the monocot–dicot divide.

#### 2.2.2. Promoter Cis-Element Analysis

Promoter analysis of the 1501 bp sequence upstream of the *RcnsLTPC* transcription start site using the PlantCARE database identified multiple cis-regulatory elements in addition to the core promoter motifs TATA-box and CAAT-box (Figure 1D). These elements included light-responsive motifs (Box 4, GT1-motif, I-box, and AT1-motif), hormone-responsive motifs (AuxRR-core, TGA-element, GARE-motif, ERE, and CGTCA-motif), and stress-related motifs (ARE, WUN-motif, MYB, and MYC). The presence of these elements indicates that multiple environmental and hormonal signals may regulate the RcnsLTPC promoter at the transcriptional level.

### 2.3. Structural and Functional Characterization of the RcnsLTPC Protein

#### 2.3.1. Physicochemical Properties and Protein Structure

The RcnsLTPC protein has a predicted molecular weight of 19.15 kDa and a theoretical isoelectric point (pI) of 9.48, classifying it as a basic protein (Table 1). An instability index of 26.16 (<40) indicates that the protein is thermostable, and a GRAVY value of −0.217 reflects the overall average hydropathicity across structurally distinct domains (detailed domain-specific hydrophobicity analysis is provided in Figure S2A). A homology-based three-dimensional structural model of RcnsLTPC was constructed using P10975.1 as the template (Figure 1E). The model achieved a MolProbity composite score of 0.62 with 97.37% of residues falling within the most favored regions of the Ramachandran plot, confirming publication-quality structural reliability.

#### 2.3.2. Conserved Domain, Signal Peptide, and Transmembrane Topology Prediction

CD-Search analysis identified a canonical nsLTP1 conserved domain (cd01960; E = 6.68 × 10^−28^) spanning residues 85–171 at the C-terminus of RcnsLTPC, with high sequence similarity to the protease inhibitor/seed storage/LTP superfamily (Pfam: PF00234) and the plant lipid transfer protein domain (SMART: SM00499) (Figure 1F). A hydrophobic cavity predicted within this domain is consistent with a role in lipid binding and transfer. Transmembrane topology prediction using TMHMM identified a single transmembrane helix spanning residues 63–85, with the N-terminal segment (residues 1–62) oriented toward the extracellular space and the C-terminal region (residues 86–175) facing the cytoplasm, supporting a type I transmembrane protein topology (Table S3). Subcellular localization prediction placed RcnsLTPC most probably in the extracellular space (probability = 0.6211), and TargetP-2.0 classified it as a secretory pathway protein with the highest reliability class (RC = 1), consistent with the transmembrane topology predictions.

### 2.4. Construction of Recombinant Vectors and Generation of Transgenic Tobacco Lines

The yeast expression vector pYES2-*RcnsLTPC* and the plant overexpression vector GV1300-*RcnsLTPC*-GFP were successfully constructed. Colony PCR and Sanger sequencing confirmed correct insertion of the target gene in the proper reading frame in both constructs (Figure S3). Following *A. tumefaciens*-mediated leaf disc transformation and multiple rounds of Hyb (25 mg·L^−1^) selection, 14 independent transgenic tobacco lines were recovered. qRT-PCR analysis identified OE-11 and OE-12 as high-expression lines based on significantly elevated *RcnsLTPC* transcript levels relative to wild type; these two lines were selected for all subsequent functional characterization.

### 2.5. Subcellular Localization of RcnsLTPC

**Figure 2:**
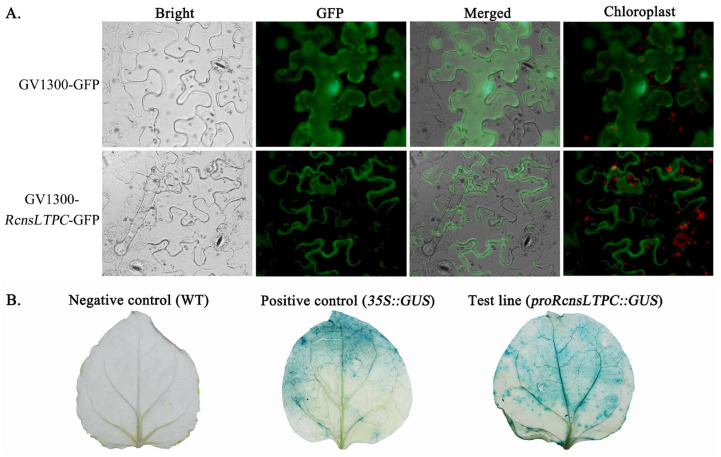
Subcellular localization of the RcnsLTPC protein in tobacco leaf epidermal cells (**A**) and GUS histochemical staining assay for transient promoter activity (**B**).

To determine the subcellular localization of RcnsLTPC, the GV1300-*RcnsLTPC*-GFP fusion construct was transiently expressed in *N. benthamiana* leaf epidermal cells via Agrobacterium-mediated infiltration (Figure 2A). In cells transformed with the empty GV1300-GFP vector, GFP fluorescence was diffusely distributed throughout the cytoplasm and nucleus. In contrast, fluorescence from the RcnsLTPC-GFP fusion protein was specifically concentrated at the cell periphery, displaying a continuous and well-defined ring pattern along the plasma membrane, with no overlap with the chloroplast autofluorescence channel. These results confirm that RcnsLTPC localizes specifically to the plasma membrane, consistent with the bioinformatic prediction of a secretory pathway protein, and provide direct cytological evidence supporting its potential role in membrane-associated lipid transport or signal recognition.

### 2.6. Verification of RcnsLTPC Promoter Activity

The transcriptional activity of the RcnsLTPC promoter was assessed by GUS histochemical staining following transient expression in *N. benthamiana* leaves (Figure 2B). No blue staining was detected in the negative control (empty vector) following chlorophyll clearing, confirming the absence of nonspecific background. Strong blue staining was observed in the positive control (35S::GUS), validating the GUS reporter system. Leaves infiltrated with the proRcnsLTPC::GUS construct similarly produced a clear blue signal, demonstrating that the cloned promoter sequence drives genuine transcriptional activity in *N. benthamiana*.

### 2.7. Salt Tolerance of RcnsLTPC-Overexpressing Yeast

#### 2.7.1. Spot Assay Under Graded NaCl Concentrations

**Figure 3:**
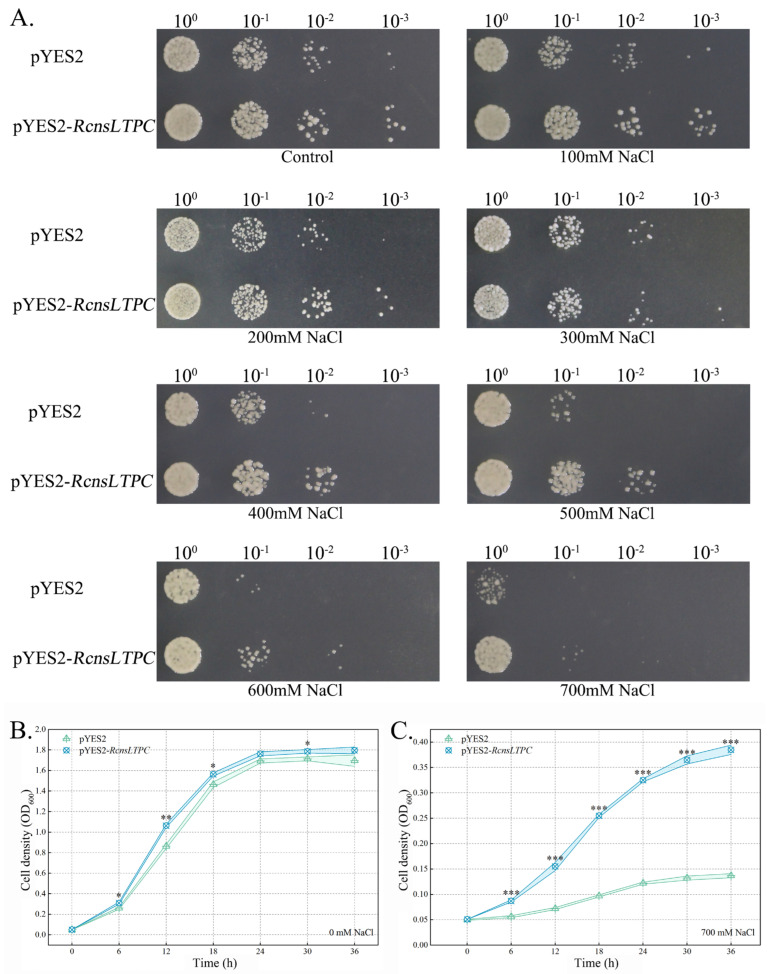
Spot assay evaluating the effect of heterologous expression of the *RcnsLTPC* gene on salt tolerance in yeast (**A**). Growth dynamics of yeast strains under different NaCl concentrations. The growth curves show changes in cell density (OD_600_) over time (h) under 0 mM NaCl (control) (**B**) and 700 mM NaCl (**C**). Asterisks indicate statistically significant differences: * *p* < 0.05, ** *p* < 0.01, *** *p* < 0.001.

Heterologous overexpression of *RcnsLTPC* conferred markedly enhanced NaCl tolerance in yeast across a broad range of salt concentrations. On SG/-Ura solid medium supplemented with 0–700 mM NaCl, the overexpression strain (pYES2-*RcnsLTPC*) and the empty vector control (pYES2) grew comparably in the absence of NaCl (Figure 3A), indicating that *RcnsLTPC* expression does not impair basal yeast viability. At NaCl concentrations between 100 and 600 mM, the overexpression strain consistently formed more colonies across all dilution series (10^0^–10^−3^), with the growth advantage becoming increasingly pronounced at higher NaCl concentrations. At 400 mM NaCl, colonies from the control strain were nearly undetectable at low inoculum densities (10^−2^ and 10^−3^), whereas the overexpression strain maintained visible colonies at 10^−1^. At 600 mM NaCl, the control strain produced only faint growth at the highest inoculum density (10^0^), while the overexpression strain retained conspicuous colony formation across dilutions from 10^0^ to 10^−2^. Even under extreme stress at 700 mM NaCl, the overexpression strain demonstrated a clear survival advantage at 10^0^ and 10^−1^ dilutions.

#### 2.7.2. Growth Kinetics Under High Salt Stress

Liquid culture experiments provided quantitative support for the salt-protective function of *RcnsLTPC* (Figure 3B,C). Under 700 mM NaCl stress, growth of the empty vector control was severely inhibited, effectively arresting after 12 h with an OD_600_ below 0.15 at 24 h. By contrast, while the overexpression strain exhibited reduced growth kinetics under the same conditions, cell density increased continuously from 6 h onward and was significantly higher than the control at all time points between 6 and 36 h (*p* < 0.001), reaching an OD_600_ of approximately 0.40 at 36 h. Under 0 mM NaCl, the overexpression strain also showed modestly but significantly higher OD_600_ values at 6, 12, and 24 h relative to the control (*p* < 0.05), suggesting that *RcnsLTPC* expression provides a slight growth advantage even under normal conditions.

### 2.8. Salt Tolerance of RcnsLTPC-Overexpressing Tobacco

#### 2.8.1. Seed Germination and Seedling Root Growth Under Salt Stress

Overexpression of *RcnsLTPC* markedly improved germination tolerance to salt stress. Across a gradient of 0–200 mM NaCl, final germination rates of WT and EV seeds declined sharply with increasing NaCl concentration, whereas OE-11 and OE-12 maintained germination rates approximately 3.8–4.2 times higher than WT at 150 mM NaCl (*p* < 0.05; Table 2). Correspondingly, OE lines exhibited significantly lower salt injury indices and higher stress tolerance indices relative to WT (Table 3). ZnO-NPs priming further enhanced germination under salt stress across all genotypes, with particularly pronounced effects in OE lines (Table 2). These results indicate a synergistic interaction between ZnO-NPs priming and *RcnsLTPC* overexpression in promoting germination under salt stress.

**Figure 4:**
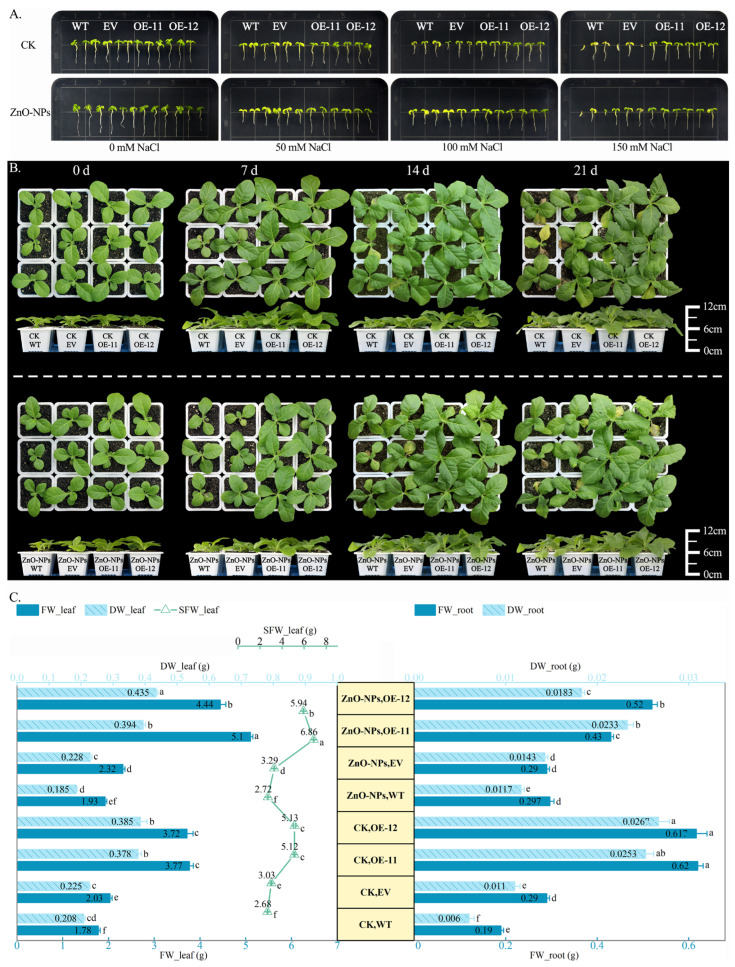
Root phenotypes of various tobacco lines primed with different concentrations of NaCl and ZnO-NPs ((**A**), scale bar = 1 cm). (**B**) Effects of *RcnsLTPC* overexpression and ZnO-NP priming on the growth dynamics of tobacco seedlings under salt stress (representative scale bar: 0–12 cm). (**C**) Biomass accumulation in tobacco under salt stress. Data are from plants under 300 mM NaCl (mean ± SE, *n* = 3). Different lowercase letters indicate significant differences (*p* < 0.05, Tukey’s test).

Root elongation in seedlings was progressively inhibited with increasing NaCl concentrations. At 150 mM NaCl, WT seedling root length was less than 0.5 cm, whereas OE maintained root lengths of 0.55–0.65 cm, significantly greater than WT (*p* < 0.05; Figure 4A). ZnO-NPs priming further alleviated salt-induced root growth inhibition, increasing root length in OE by approximately 15–20% relative to unprimed counterparts at 100 and 150 mM NaCl, with the synergistic benefit being more pronounced in OE than in WT.

#### 2.8.2. Growth Phenotype and Biomass Accumulation in Mature Plants

*RcnsLTPC* overexpression substantially improved the growth performance of mature plants under prolonged salt stress. Over a 21-day exposure to 300 mM NaCl, WT and EV plants showed basal leaf chlorosis from day 7, progressing to extensive yellowing by day 14 (Figure 4B), while OE exhibited markedly attenuated chlorosis and retained a greater proportion of green, photosynthetically active leaves at day 21. Primed OE plants consistently displayed the best overall morphology, with superior shoot height, leaf area, and biomass throughout the stress period.

At day 21, OE-11 and OE-12 leaf fresh weights exceeded those of WT by more than 2-fold, with root fresh weight similarly elevated (*p* < 0.05; Figure 4C). OE plants also maintained significantly higher RWC and lower WSD, alongside a markedly increased R/S relative to WT (Table S4). The combination of ZnO-NPs priming and *RcnsLTPC* overexpression produced the most pronounced improvements in all biomass parameters, with primed OE-11 plants achieving values significantly exceeding all other treatment groups.

#### 2.8.3. Root Architecture Analysis

**Figure 5:**
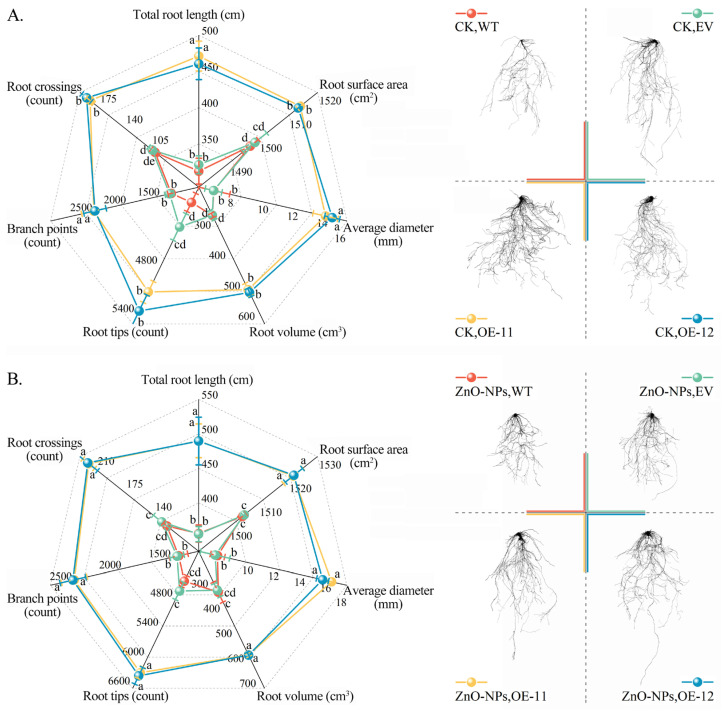
Effects of *RcnsLTP*C overexpression and ZnO-NP priming on tobacco root architecture under 300 mM NaCl stress for 21 days. Radar charts and representative root-scanning images display root morphology without priming (CK) (**A**) and after ZnO-NP priming (**B**). Data points in the radar charts represent the mean of ≥3 biological replicates. Different lowercase letters at the vertices indicate significant differences (*p* < 0.05, Tukey’s test) among different genotypes for the same parameter.

In unprimed plants following 21 days of salt treatment, *RcnsLTPC* overexpression significantly promoted root morphological development under salt stress (Figure 5). Relative to WT, unprimed OE-11 showed approximately 51% greater total root length, 83% larger root volume, and 2.14-fold more branching points (*p* < 0.05), with OE-12 displaying comparable trends. ZnO-NPs priming further amplified these improvements, with the synergistic effects most pronounced in root volume and branching point number; primed OE lines consistently exceeded both primed and unprimed WT across all root morphological parameters.

### 2.9. Oxidative Stress Responses Under Salt Stress

#### 2.9.1. Membrane Injury Indicators: MDA Content and REC

**Figure 6:**
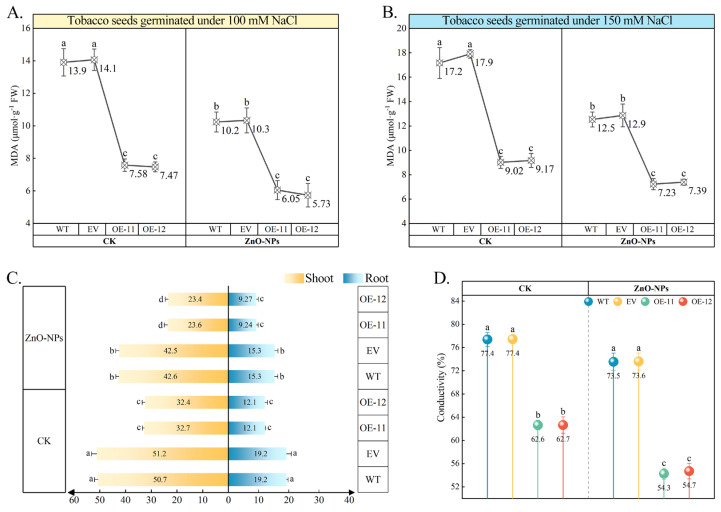
Effects of *RcnsLTPC* overexpression and ZnO-NP priming on MDA content and membrane integrity in tobacco under salt stress. Shown are MDA content during seed germination under different NaCl concentrations (**A**,**B**), MDA content in leaves and roots at the adult plant stage (**C**), and relative electrical conductivity in leaves (**D**). Data are mean ± SD (*n* = 3); different lowercase letters indicate significant differences (*p* < 0.05, Tukey’s test).

*RcnsLTPC* overexpression effectively alleviated salt stress-induced membrane lipid peroxidation. MDA content, a marker of lipid peroxidation, was significantly reduced in OE relative to WT at both germination and mature stages (Figure 6A–D). Under salt stress, OE consistently exhibited approximately 45–47% lower MDA levels than WT during germination, and approximately 35–37% lower levels in mature plant leaves and roots (*p* < 0.05). ZnO-NPs priming further reduced MDA content in all genotypes, with the greatest additional reduction observed in OE.

Leaf REC followed the same trend as MDA (Figure 6D). OE exhibited approximately 19% lower REC than WT under unprimed salt stress conditions, confirming preserved plasma membrane integrity (*p* < 0.05). ZnO-NPs priming further reduced REC in OE, with primed OE showing significantly lower values than either primed WT or unprimed OE.

#### 2.9.2. Histochemical Staining and Quantification of ROS Accumulation

**Figure 7:**
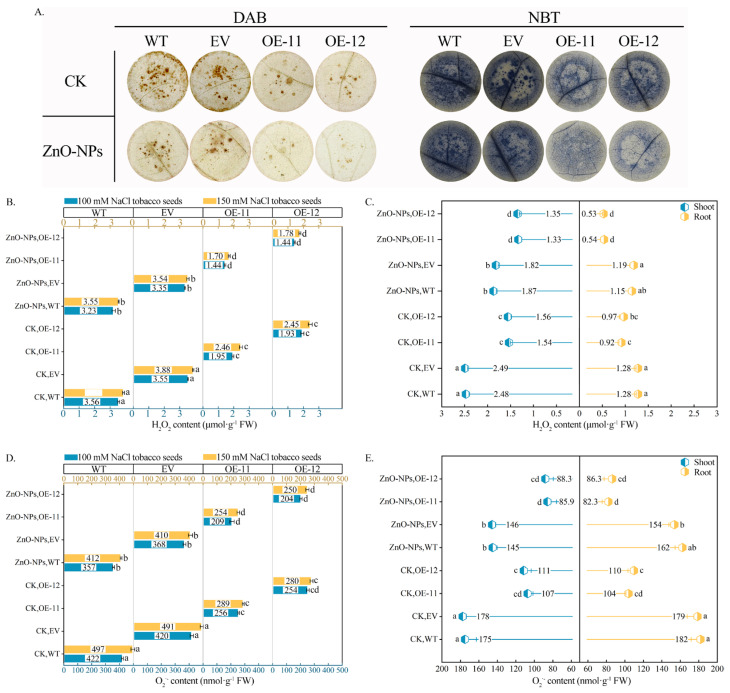
Histochemical staining of ROS accumulation: DAB staining detects H_2_O_2_ (brown precipitate), while NBT staining detects O_2_^·−^ (blue formazan precipitate) (**A**), effects of *RcnsLTPC* overexpression and ZnO-NPs priming on reactive oxygen species (H_2_O_2_ and O_2_^−^) accumulation in tobacco under salt stress. H_2_O_2_ (**B**) and O_2_^·−^ (**C**) contents during seed germination; H_2_O_2_ (**D**) and O_2_^·−^ (**E**) contents in leaves and roots at the adult plant stage. Data are presented as mean ± SD (*n* = 3). Different lowercase letters indicate significant differences among treatments (*p* < 0.05, Tukey’s test).

DAB and NBT staining were used to visualize the spatial distribution of H_2_O_2_ and O_2_^·−^, respectively (Figure 7A). Under unprimed conditions, WT and EV leaves showed intense brown DAB staining and extensive deep-blue NBT staining, indicative of high levels of both ROS. In contrast, OE-11 and OE-12 leaves exhibited markedly attenuated staining signals, with only sparse and faint deposits. Following ZnO-NPs priming, the relative pattern of staining intensity across genotypes was preserved, but staining in OE was further diminished, suggesting an additive effect of nanoparticle priming and *RcnsLTPC* overexpression on ROS scavenging.

Quantitative ROS analysis corroborated the histochemical staining results. During germination under 100 mM NaCl, H_2_O_2_ and O_2_^·−^ levels in OE seeds were approximately 45% and 39% lower than in WT, respectively (*p* < 0.05; Figure 7B,D), with comparable reductions observed at 150 mM NaCl. In mature plant leaves and roots, OE similarly maintained 37–44% lower H_2_O_2_ and O_2_^·−^ levels than WT (Figure 7C,E). The synergistic effect of ZnO-NPs priming and *RcnsLTPC* overexpression on ROS scavenging was particularly pronounced, with primed OE producing substantially greater ROS reductions than either treatment alone, an effect consistent across both germination and mature plant stages.

#### 2.9.3. Antioxidant Enzyme Activities

**Figure 8:**
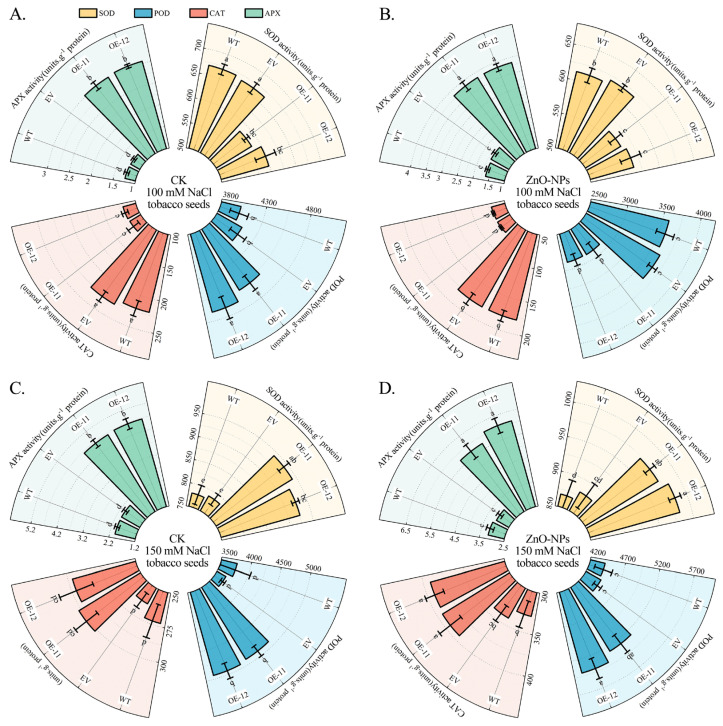
Effects of *RcnsLTPC* overexpression and ZnO-NP priming on antioxidant enzyme activities in tobacco under salt stress. Activities of antioxidant enzymes in germinating seeds under 100 mM (**A**,**B**) and 150 mM (**C**,**D**) NaCl stress, without (CK) or with ZnO-NP priming. Data are presented as mean ± SD (*n* = 3). Different lowercase letters indicate significant differences (*p* < 0.05, Tukey’s test).

*RcnsLTPC* overexpression enhanced antioxidant enzyme activities in a stress intensity-dependent and enzyme-specific manner (Figure 8). During germination under mild salt stress (100 mM NaCl), OE exhibited a selective enzyme activation profile, with significant increases in APX (by 26.82%) and POD (by 14.20%) activities and concurrent reductions in SOD (by approximately 10–13%) and CAT (by approximately 40%) activities (*p* < 0.05). Under more severe stress (150 mM NaCl), a comprehensive upregulation of all four antioxidant enzymes was observed in OE relative to WT: SOD, POD, CAT, and APX activities were elevated by 20.80%, 30.09%, 10.63%, and 132.66%, respectively, indicative of a fully activated enzymatic antioxidant defense network.

In mature plant leaves under 300 mM NaCl, OE-11 showed increases of 43.18%, 28.03%, 46.17%, and 119.25% in SOD, POD, CAT, and APX activities, respectively, relative to WT (*p* < 0.05). Even greater enhancements were observed in roots, where POD, CAT, and APX activities were elevated by 45.03%, 64.86%, and 174.65% (Figure S4), respectively. ZnO-NPs priming further amplified antioxidant enzyme activities in OE; notably, APX activity in primed OE-11 leaves reached 3.25-fold that of WT, representing the most pronounced synergistic response among all enzymes measured.

### 2.10. Ion Homeostasis and Candidate Gene Expression Under Salt Stress

#### 2.10.1. Na^+^/K^+^ Content and Ionic Homeostasis

*RcnsLTPC* overexpression significantly improved ion homeostasis in both leaves and roots of salt-stressed tobacco plants. Under 300 mM NaCl, leaf Na^+^ content in unprimed OE (~41.60 mg·kg^−1^) was only 40.39% of that in WT (103.00 mg·kg^−1^), while K^+^ content (~35.33 mg·kg^−1^) was 40.64% higher than in WT. Consequently, the Na^+^/K^+^ ratio was dramatically reduced from 4.23 in WT to 1.19 in OE (a 71.87% decrease; *p* < 0.05). Similar trends were observed in roots, where OE-11 and OE-12 showed Na^+^ reductions of 43.85% and 41.32%, respectively, K^+^ increases of approximately 90.95%, and Na^+^/K^+^ ratios approximately 70.32% lower than WT.

ZnO-NPs priming partially alleviated ion toxicity in WT plants, reducing leaf Na^+^ by 28.04% and lowering the Na^+^/K^+^ ratio from 4.23 to 3.27. The combination of ZnO-NPs priming and *RcnsLTPC* overexpression achieved the most favorable ionic balance, with primed OE-11 exhibiting Na^+^/K^+^ ratios in both leaves and roots approximately 70–82% lower than WT (*p* < 0.05; Table 4), demonstrating the strongest ion homeostasis maintenance among all treatment groups.

#### 2.10.2. Expression of Salt Stress-Related Candidate Genes

To further elucidate the molecular basis by which *RcnsLTPC* enhances salt tolerance, the expression levels of key stress-responsive genes were examined by qRT-PCR. Following 21 days of 300 mM NaCl treatment, OE showed significant upregulation of the antioxidant-related genes *NtSOD*, *NtPOD*, and *NtCAT* by 1.76-, 1.44-, and 1.36-fold relative to WT, respectively. The vacuolar Na^+^/H^+^ antiporter gene *NtNHX1* and the high-affinity K^+^ transporter gene *NtHKT1* were upregulated 1.73- and 1.60-fold, respectively (*p* < 0.05; Figure S5). The coordinated transcriptional upregulation of these five candidate genes demonstrates that *RcnsLTPC* overexpression simultaneously activates both the antioxidant defense machinery and the ion compartmentalization and transport network, revealing a multi-pathway transcriptional mechanism underlying the enhanced salt tolerance conferred by *RcnsLTPC*.

## 3. Discussion

**Figure 9:**
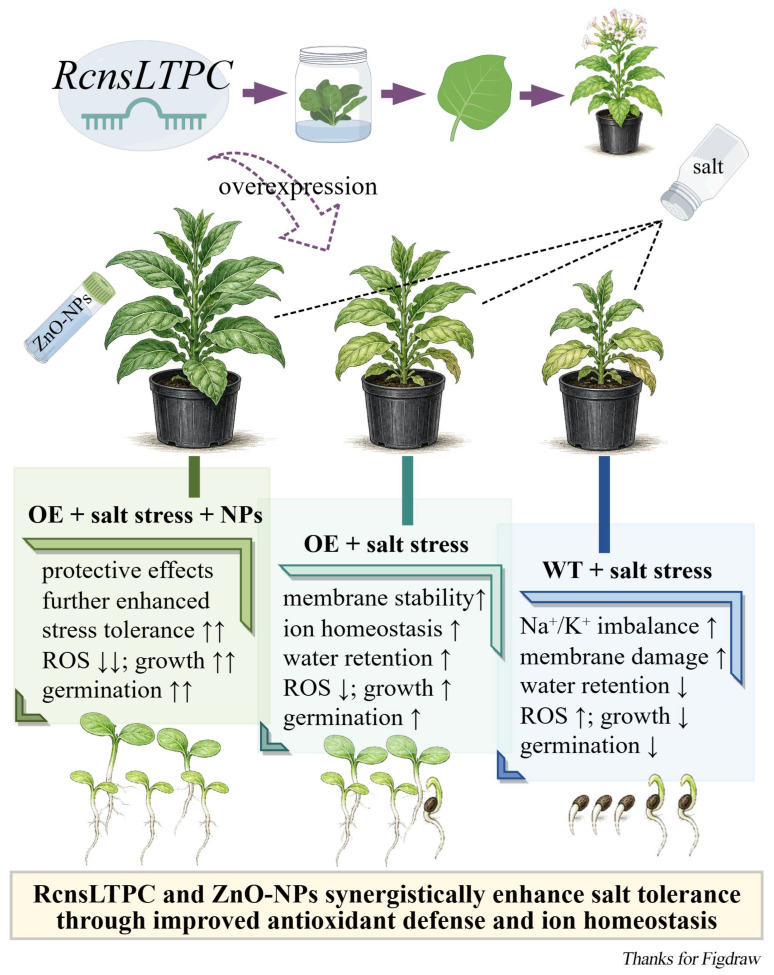
Proposed model of *RcnsLTPC*-mediated salt tolerance in tobacco with ZnO-NPs priming. WT plants show salt-induced ion imbalance, ROS accumulation, and growth inhibition. RcnsLTPC overexpression improves membrane stability, ion homeostasis, and ROS scavenging. ZnO-NPs priming further enhances these protective effects and promotes salt tolerance.

This study provides the first evidence that *RcnsLTPC* enhances salt tolerance in yeast and transgenic tobacco, with further improvement observed upon ZnO-NPs priming. *RcnsLTPC* encodes a plasma membrane-localized protein that confers robust salt-stress tolerance through three coordinated mechanisms: enhanced antioxidant enzyme activity, maintenance of Na^+^/K^+^ homeostasis, and optimization of root architecture. These findings highlight a previously unexplored role of nsLTPs in castor and provide proof of concept for integrating nanopriming with functional gene enhancement in crop improvement (Figure 9).

### 3.1. Phylogenetic Position and Promoter Regulatory Features of RcnsLTPC

Phylogenetic analysis revealed that *RcnsLTPC* clusters with three other paralogous proteins in the castor genome (RcnsLTPA, RcnsLTPB, and RcnsLTPD) to form a monophyletic clade with 100% bootstrap support, which is consistent with the possibility that these four genes arose from a recent tandem duplication event within the castor genome. Tandem gene duplication is recognized as a key mechanism driving the expansion of the plant nsLTP family. It has been systematically documented in species such as *Brassica* [20] and barley [21]. Notably, this castor-specific clade exhibits a closer evolutionary relationship with LTP proteins from monocotyledonous species, suggesting that *RcnsLTPC* may retain certain conserved functional features. Comprehensive analyses of nsLTP phylogeny have corroborated such evolutionary conservation across both monocots and dicots and have been proposed to be associated with ancient defense mechanisms that underpin plant responses to abiotic stresses [22].

The composition of cis-acting elements within a promoter is critical for understanding gene regulatory networks. In this study, the *RcnsLTPC* promoter was found to be enriched with light-responsive elements (Box 4, GT1-motif, I-box), multiple hormone-responsive elements (AuxRR-core, GARE-motif, ERE, CGTCA-motif), and abiotic stress–responsive elements (MYB and MYC binding sites, ARE, WUN-motif). This complex arrangement of cis-elements resembles previously reported nsLTP promoters, such as those in sugarcane [23], suggesting that multiple signaling pathways coordinately regulate *RcnsLTPC* expression. Notably, the presence of MYB and MYC binding sites is of particular interest, as these transcription factors serve as central regulatory nodes in the ABA signaling pathway and play critical roles in plant responses to drought and salt stress [24]. The identification of a methyl jasmonate-responsive element (CGTCA-motif) further indicates potential cross-talk between *RcnsLTPC* expression and JA-mediated defense signaling. Functional validation using a GUS reporter assay confirmed the transcriptional activity of the cloned promoter in *N. benthamiana*, providing a solid foundation for subsequent applications.

### 3.2. Structural Features and Subcellular Localization of RcnsLTPC

Unlike most nsLTPs, which are strongly hydrophobic (GRAVY > 0), RcnsLTPC exhibits a global GRAVY value of −0.217. However, this value reflects the combined contributions of structurally distinct regions rather than a uniform physicochemical property of the entire sequence. Local hydrophobicity analysis using the Kyte–Doolittle algorithm (Figure S2A) reveals pronounced domain-specific variation. The N-terminal region (residues 1–62) is predominantly hydrophilic, with scores largely below zero. By contrast, the central segment (residues 63–85) displays a sharp hydrophobic peak of approximately +3.0, consistent with the transmembrane helix predicted by TMHMM. The C-terminal nsLTP1 domain (residues 85–171), which contains the conserved eight-cysteine motif, shows a mixed hydrophobic/hydrophilic profile with moderate fluctuations around zero, rather than the sustained high hydrophobicity typical of many classical lipid-transfer nsLTPs. This domain organization suggests that RcnsLTPC may differ from canonical nsLTPs in its lipid-binding properties and may instead be more closely associated with signal recognition and transduction than with direct lipid carriage [25]. This interpretation is further supported by analogous hydrophilic nsLTPs reported in *A. thaliana* [26] and rice [27], both of which have been linked to stress responses.

TMHMM analysis predicted a transmembrane helix spanning residues 63–85 of RcnsLTPC, characteristic of a type I transmembrane protein, and TargetP-2.0 classified it with the highest confidence as a secretory pathway protein. Subcellular localization experiments using transient expression of a GFP fusion in *N. benthamiana* epidermal cells directly confirmed that RcnsLTPC localizes specifically to the plasma membrane, in strong agreement with bioinformatic predictions. Plasma membrane localization is functionally significant for nsLTPs, as the plasma membrane constitutes a major interface for sensing and responding to external osmotic stress. Proteins localized here may participate in the generation and transduction of lipid-based signals and may influence membrane lipid composition, thereby contributing to membrane stability under salt stress [28].

Previous studies have demonstrated that plasma membrane–localized nsLTPs can modulate H^+^-ATPase and Na^+^/H^+^ antiporter activities by regulating phosphatidylinositol (PI) phosphorylation, thereby participating in ABA signaling [29]. This aligns with our finding that the RcnsLTPC promoter contains ABA-responsive MYB/MYC elements, supporting the possibility that *RcnsLTPC* may contribute to ABA-mediated salt stress responses via plasma membrane lipid remodeling. Furthermore, evidence for synergistic interactions between pea LTPs and ABA further supports the functional model in which plasma membrane-localized nsLTPs intersect with hormonal signaling pathways [30].

It is worth noting that TargetP-2.0 classifies RcnsLTPC as a secretory pathway protein (extracellular, *p* = 0.62), and transmembrane topology analysis predicts a cytoplasmic C-terminus. Since the GFP tag in our construct is fused to the C-terminus, the fluorescent reporter would be expected to reside on the cytoplasmic face of the plasma membrane, which is fully consistent with the continuous plasma membrane ring pattern observed by confocal microscopy. The TargetP-2.0 result is therefore interpreted as evidence for secretory pathway-mediated trafficking to the plasma membrane rather than extracellular secretion; indeed, several nsLTP family members are known to utilize the secretory pathway before achieving stable plasma membrane residence, including GPI-anchored nsLTPs detected at the outer face of the plasma membrane [17]. Co-localization with an established plasma membrane marker such as AtPIP2A would provide definitive confirmation and represents an important direction for future investigation.

### 3.3. Functional Validation of RcnsLTPC in Yeast Under Salt Stress

The heterologous expression system in Saccharomyces cerevisiae is a classical eukaryotic model for assessing plant gene–mediated salt tolerance, with a well-characterized genetic background and partially conserved mechanisms for osmotic and ion homeostasis shared with plants [31]. In the present study, across a broad NaCl concentration range of 100–700 mM, *RcnsLTPC*-overexpressing yeast exhibited pronounced growth advantages in both spot assay and liquid culture systems, with tolerance increasingly evident under higher stress intensities. Under extreme stress at 700 mM NaCl, the OD_600_ of overexpression strains reached approximately 0.40 at 36 h, whereas control strains showed almost complete growth arrest. This magnitude of tolerance is comparable to previously reported nsLTP family genes in yeast; for example, heterologous expression of *HcnsLTP111* from *Hibiscus cannabinus* markedly enhanced survival and mitigated oxidative damage under salt stress [32], and overexpression of *CmnsLTP6.9* from *Castanea mollissima* improved ROS scavenging and regulated membrane lipid metabolism, thereby increasing osmotic stress tolerance [33].

Interestingly, *RcnsLTPC* overexpression also slightly promoted yeast proliferation under non-stress conditions, suggesting that the protein may positively modulate basal cellular lipid metabolism and membrane function, although the precise mechanisms remain to be elucidated [32,33]. Overall, the yeast functional assays provide strong evidence that *RcnsLTPC* directly contributes to salt tolerance, rather than acting solely through indirect regulation of downstream genes, and lay a solid foundation for subsequent molecular characterization in planta.

### 3.4. Salt Tolerance Mechanisms in RcnsLTPC-Overexpressing Tobacco

#### 3.4.1. Salt Tolerance During Germination and Early Seedling Growth

The seed germination stage is one of the most sensitive periods in the plant life cycle to salt stress, during which damage is closely associated with impaired water uptake due to high osmotic potential and Na^+^ toxicity [34]. In this study, *RcnsLTPC*-OE-11 and OE-12 exhibited germination rates under 150 mM NaCl that were approximately 3.8–4.2 times higher than those of the wild type, indicating a pronounced protective effect of *RcnsLTPC* during the germination stage. Similar growth-promoting effects under stress conditions have been reported for nsLTP overexpression in other species, including *SiLTP* in millet [35] and *SpLTP1* in desert plants [36]. However, the magnitude of enhancement varies depending on the species and stress intensity.

Roots of seedlings serve as critical organs for perceiving and responding to ionic toxicity under salt stress. Under 150 mM NaCl, root elongation in WT was nearly arrested, whereas OE maintained significant root growth advantages. Analysis of mature root systems further revealed that overexpression lines outperformed WT plants in total root length (increased by ~51%), root volume (increased by ~83%), and number of branching points (increased by ~2.14-fold). The increased root system complexity likely confers a larger absorptive surface area and enhanced capacity for water and mineral nutrient acquisition [37]. These results suggest that the salt tolerance conferred by *RcnsLTP*C is, at least in part, associated with optimization of root architecture and improved resource acquisition under saline conditions.

#### 3.4.2. Oxidative Stress Defense Mechanisms

Excessive accumulation of ROS under salt stress is a key driver of cellular oxidative damage, as ROS-mediated lipid peroxidation directly compromises membrane integrity and contributes to salt-induced lethality [38]. In this study, oxidative damage parameters were systematically evaluated in *RcnsLTP*C-OE during both germination and mature plant stages. Consistently, OE exhibited a significant reduction in H_2_O_2_ and O_2_^·−^ accumulation (by 36–46%), accompanied by decreased MDA content and maintenance of lower membrane conductivity. These results indicate that *RcnsLTPC* modulates the ROS–membrane damage axis across both germination and vegetative stages, suggesting a broad protective role against oxidative stress.

Regarding antioxidant enzyme activities, *RcnsLTP*C overexpression exhibited a clear stress–intensity–dependent regulatory pattern. Under mild salt stress (100 mM NaCl), OE preferentially activated the APX and POD pathways, while SOD and CAT activities were relatively reduced. When stress intensity increased to 150 mM NaCl, activities of all four enzymes were significantly upregulated (SOD by 20.80%, POD by 30.09%, CAT by 10.63%, and APX by 132.66%), forming a comprehensive enzymatic antioxidant defense network. This graded activation pattern is biologically significant: APX specifically scavenges H_2_O_2_ in chloroplasts and the cytosol, and its preferential activation effectively protects the photosynthetic apparatus from oxidative damage. SOD catalyzes the dismutation of O_2_^·−^ into H_2_O_2_, which requires CAT for subsequent removal; the delayed activation of CAT may reflect the cell’s fine-tuned perception of oxidative stress intensity [39]. Such a stress intensity–dependent antioxidant response is consistent with efficient cellular resource utilization [38]. Furthermore, transcript levels of *NtSOD*, *NtPOD*, and *NtCAT* were significantly upregulated in OE, suggesting that nsLTPs may modulate antioxidant enzyme gene expression through redox signaling cascades, consistent with a model in which plasma membrane–localized *RcnsLTPC* may regulate downstream signaling via dynamic modulation of membrane lipids [40,41].

#### 3.4.3. Regulation of Ion Homeostasis

Maintaining a dynamic balance of the intracellular Na^+^/K^+^ ratio is a central physiological basis for plant salt tolerance [31]. In this study, under 300 mM NaCl stress, *RcnsLTPC* overexpression markedly reduced Na^+^ accumulation in both leaves and roots of tobacco, while simultaneously increasing K^+^ content, resulting in a substantial decrease in the Na^+^/K^+^ ratio (71.87% reduction in leaves and 70.32% reduction in roots). The expression of candidate genes mechanistically supports this phenotypic effect: transcription levels of the vacuolar Na^+^/K^+^ antiporter gene *NtNHX1* and the high-affinity K^+^ transporter gene *NtHKT1* were significantly upregulated in OE, in strong agreement with the measured ion content. These findings are consistent with previous reports showing that SOS1 overexpression maintains K^+^/Na^+^ balance [42] and that *AtHKT1* promotes overall K^+^ homeostasis in plants [43].

Vacuolar sequestration of Na^+^ mediated by *NHX1* and selective K^+^ uptake mediated by *HKT1* represent two of the most extensively studied pathways for maintaining ion homeostasis in plant salt tolerance. Overexpression of *RcnsLTP*C simultaneously activated the key genes in both pathways, suggesting that *RcnsLTPC* overexpression is associated with a broader transcriptional response encompassing both ion sequestration and selective uptake pathways. However, whether *RcnsLTPC* directly regulates these networks as an upstream component remains to be experimentally established. Although the molecular mechanisms by which nsLTPs participate in ion transport regulation remain unclear, one possibility is that they influence the membrane localization, activity, or transcription of relevant transporters via plasma membrane PA signaling—PA has been shown to promote the localization of *MKK7*/*MKK9* to the plasma membrane and activate stress signaling cascades [44]. This hypothesis warrants further validation through lipidomic profiling and protein–protein interaction analyses in future studies.

### 3.5. Synergistic Enhancement Mechanism of ZnO-NPs Priming and RcnsLTPC Overexpression

Nanoparticle-based seed priming is an emerging seed treatment strategy that can improve germination performance and stress resilience by pre-activating metabolic and transcriptional programs within the seed [45]. ZnO-NPs have attracted considerable attention because they can sustainably release Zn^2+^, modulate ROS signaling, and activate defense-related enzymatic systems [19]. However, synergistic studies combining nanoparticle priming with genetic engineering strategies remain very limited at present, and this study provides systematic experimental evidence for this interaction.

This study found that ZnO-NPs priming (50 mg·L^−1^) and *RcnsLTPC* overexpression exhibited a pronounced synergistic effect across multiple parameters, including germination rate, root architecture, biomass accumulation, MDA content, ROS levels, antioxidant enzyme activities, and Na^+^/K^+^ homeostasis. The combined treatment produced a substantially greater effect than the simple sum of the two individual treatments. Taking APX activity as an example, APX activity in the leaves of the primed OE-11 plants reached 3.25-fold that of the WT. In contrast, ZnO-NPs priming alone in WT or OE alone each increased APX activity by only approximately 1.5- to 2.0-fold, suggesting a positive interaction between the two treatments.

The mechanistic basis of this synergy likely involves multiple non-mutually exclusive pathways, including Zn^2+^-mediated cofactor supplementation for antioxidant enzymes, ZnO-NPs-induced transcriptional priming, and Zn^2+^-assisted membrane stabilization [46,47,48]. However, these interpretations remain speculative, and the precise contributions of nanoparticle priming and gene overexpression to the observed synergy have yet to be formally dissected. The phenotypic and physiological data generated in this study nevertheless provide strong functional evidence for the existence of such synergy and offer proof of concept for an integrated “nanotechnology plus genetic engineering” strategy.

### 3.6. Limitations and Prospects

The functional validation system in this study relied on tobacco as a heterologous host, which limits the direct extrapolation of these conclusions to castor production practices. In addition, the native lipid substrates of RcnsLTPC and its direct protein–protein interactions with downstream signaling components remain unclear. Future studies should focus on the following aspects: (1) validating the endogenous function of *RcnsLTPC* in castor through CRISPR/Cas9-mediated knockout or RNA interference; (2) using lipidomics to clarify how *RcnsLTPC* overexpression or knockout affects membrane lipid composition; (3) identifying *RcnsLTPC-*interacting proteins by Co-IP or Y2H assays to precisely locate its molecular targets within the salt-stress signaling network; and (4) evaluating the practical applicability of the combined ZnO-NPs priming and genetic improvement strategy under field conditions, thereby providing a scientific basis for the development of salt-tolerant castor cultivars.

In summary, this study provides evidence that *RcnsLTP*C is a positive regulator of salt tolerance. Its encoded protein localizes to the plasma membrane. It confers broad-spectrum, multilayered salt-stress tolerance by coordinating the enhancement of antioxidant defense, maintenance of ion homeostasis, and optimization of root architecture. The observed synergistic enhancement between ZnO-NPs priming and *RcnsLTPC* overexpression provides proof of concept for an integrated “nanotechnology plus genetic engineering” strategy. It may hold promise for practical application and translational development.

## 4. Materials and Methods

### 4.1. Plant Materials and Growth Conditions

This study used the castor cultivar ‘Fenbi 7’, provided by Shanxi Jingzuo Castor Technology Co., Ltd. (Taiyuan, China), as the material for *RcnsLTP*C cloning. Seeds of wild-type (WT) *Nicotiana tabacum* and *Nicotiana benthamiana* were maintained in our laboratory. Before use, all seeds were surface-sterilized with 5% sodium hypochlorite for 10 min and rinsed 5 times with sterile water.

ZnO-NPs (average diameter 30 ± 10 nm, purity ≥ 99.9%, Cat. No. RH449605; Ron Reagent, Shanghai, China) were used in this study. The physicochemical characterization of this ZnO-NP material, including FT-IR, XRD, and DLS analyses, has been reported previously in our earlier publication [19]. The priming concentration was set at 1000 mg·L^−1^ for castor seeds, based on previously optimized conditions [19,49], and at 50 mg·L^−1^ for tobacco seeds based on preliminary experiments. Castor seeds were primed in the dark at 25 ± 1 °C and 125 rpm for 24 h, whereas tobacco seeds were primed for 12 h. After priming, seeds were restored to their initial moisture content.

Castor seeds were germinated as described previously [19]. Germinating seed endosperm tissue was harvested for subsequent RNA and genomic DNA extraction. Tobacco seeds were sown on MS solid medium containing 0, 100, 150, or 200 mM NaCl and incubated in a growth chamber under a 12 h light/12 h dark photoperiod at 25 ± 1 °C. Each treatment included three biological replicates.

### 4.2. Cloning of RcnsLTPC and Its Promoter, and Generation of Transgenic Lines

Information on the GV1300-GFP, pYES2, and pBI121-GUS vectors used in this study is provided in Figure S1. Based on the unigene sequence assembled from castor transcriptome data [19], the open reading frame (ORF) of *RcnsLTPC* was predicted using NCBI ORF Finder, and full-length amplification primers were designed (Table S1). Total RNA was extracted from germinating castor seed endosperm using the Plant RNA Kit (R6827; OMEGA, Norcross, GA, USA). RNA integrity was verified by agarose gel electrophoresis, and concentration and purity were assessed spectrophotometrically (A260/A280 ratio of 1.8–2.0). First-strand cDNA was synthesized using the ReverTra Ace qPCR RT Master Mix with gDNA Remover (FSQ-301; TOYOBO, Osaka, Japan). The full-length coding sequence of *RcnsLTPC* was amplified from cDNA using KOD-Plus-Neo high-fidelity DNA polymerase (TOYOBO, Osaka, Japan), purified, ligated into the pEASY-Blunt vector, and transformed into Escherichia coli DH5α. Positive clones were screened by colony PCR using M13 universal primers and confirmed by sequencing.

For the plant overexpression construct, the verified *RcnsLTPC* sequence was amplified using primers containing *Sal*I and *Bam*HI restriction sites, and the product was inserted into the *Sal*I/*Bam*HI-digested GV1300-GFP vector to generate GV1300-*RcnsLTPC*-GFP. The recombinant plasmid was introduced into *Agrobacterium tumefaciens* EHA105 for stable tobacco transformation and transient expression in *N. benthamiana*. Transgenic tobacco plants were generated using the leaf disc method, and positive transformants were selected on MS medium supplemented with 25 mg·L^−1^ hygromycin B (Hyb). Transgenic lines were verified by genomic PCR using the primer pair *RcnsLTPC*-*Sal*I-F/*RcnsLTPC*-*Bam*HI-R (Table S1), which amplifies a 1280 bp fragment spanning the *RcnsLTPC* insert and part of the GFP sequence. Two independent overexpression lines (OE-11 and OE-12) with confirmed transgene integration were selected for further analysis. In parallel, a yeast overexpression construct, pYES2-*RcnsLTPC*, was generated by *Bam*HI/*Xba*I double digestion and ligation into the pYES2 vector, and subsequently transformed into *Saccharomyces cerevisiae* INVSc1 for functional validation.

For promoter analysis, genomic DNA extracted from castor seeds was used as the template, and primers were designed based on the 1501 bp region upstream of the *RcnsLTPC* transcription start site (Table S1). The amplified fragment was cloned into the pMD18-T vector and confirmed by sequencing. Cis-acting regulatory elements were predicted using PlantCARE and visualized with TBtools (v2.370). To verify promoter activity, the proRcnsLTPC::GUS construct, the 35S::GUS positive control, and the empty-vector negative control were transiently expressed in *N. benthamiana* leaves by agroinfiltration, followed by GUS histochemical staining.

### 4.3. Bioinformatic Analysis

Homologous *RcnsLTPC* sequences were retrieved from the NCBI RefSeq database, aligned using DNAMAN, and used to construct a neighbor-joining phylogenetic tree in MEGA 7 with 1000 bootstrap replicates. Conserved domains were identified using the NCBI Conserved Domain Search (CD Search). Protein physicochemical properties, including molecular weight, theoretical isoelectric point, instability index, aliphatic index, and grand average of hydropathicity (GRAVY), were predicted using ProtParam (ExPASy). Hydrophobicity profiling was performed with ProtScale using the Kyte–Doolittle algorithm. Transmembrane helices were predicted with TMHMM 2.0, and signal peptides were identified using SignalP 6.0. N-glycosylation sites were predicted with NetNGlyc 1.0, and post-translational modification sites (acetylation, ubiquitination, methylation, and hydroxylation) were predicted using MusiteDeep. Secondary structure was predicted with SOPMA, and the tertiary structure model was constructed by homology modeling using SWISS-MODEL. Subcellular localization was predicted using DeepLoc 2.0 and TargetP 2.0. Promoter cis-acting elements were analyzed using PlantCARE. The URLs and detailed information for all online tools are provided in Table S2.

### 4.4. Subcellular Localization Analysis

To determine the subcellular localization of *RcnsLTPC*, *A. tumefaciens* GV3101 harboring GV1300-*RcnsLTPC*-GFP or the empty GV1300-GFP vector was cultured overnight, harvested by centrifugation, and resuspended in infiltration buffer (10 mM MgCl_2_, 10 mM MES, pH 5.6) to an OD_600_ of approximately 1.5. Acetosyringone was added to a final concentration of 200 µM, and the suspension was incubated at room temperature for 3 h before infiltration. The abaxial surface of N. benthamiana leaves (8–10 leaf stage) was infiltrated using a needleless syringe. Infiltrated plants were kept in the dark for 12 h and then transferred to standard growth conditions for an additional 48 h. The lower epidermis of the infiltrated leaves was carefully peeled off, and the fluorescence signal of the *RcnsLTPC*-GFP fusion protein was observed using a fluorescence microscope (Axioscope 5; ZEISS, Jena, Germany) with an excitation wavelength of 488 nm. The subcellular localization pattern was compared with that of the GFP-only control.

### 4.5. Functional Analysis of RcnsLTPC in Yeast

The recombinant plasmid pYES2-*RcnsLTPC* and the empty pYES2 vector were separately transformed into *S. cerevisiae* INVSc1 using the PEG/LiAc method. The transformants were plated on SG/-Ura solid selection medium and incubated at 29 °C for 48–96 h. A single colony was inoculated into SG/-Ura liquid medium and cultured at 29 °C and 200 rpm until the OD_600_ reached approximately 0.5. Equal volumes of the resulting culture and 50% glycerol were then mixed, and the suspension was stored at −4 °C.

To evaluate the effect of *RcnsLTPC* on yeast salt tolerance, the cultured yeast suspension was serially diluted to OD_600_ values of 0.05, 0.005, 0.0005, and 0.00005, and 5 μL of each dilution was spotted onto SG/-Ura solid medium containing 0, 100, 200, 300, 400, 500, 600, or 700 mM NaCl. The plates were incubated at 29 °C for 3–4 d, and colony growth was recorded. For growth-curve analysis, yeast cells with an OD_600_ of approximately 0.05 were resuspended in SG/-Ura liquid medium containing 700 mM NaCl and 1% raffinose, cultured at 29 °C and 200 rpm for 36 h, and OD_600_ was measured at 0, 6, 12, 18, 24, 30, and 36 h.

### 4.6. Growth and Salt-Stress Treatment of Transgenic Tobacco

To evaluate the effect of *RcnsLTPC* overexpression on salt tolerance in tobacco, seeds of the WT, EV, OE-11, and OE-12 lines were surface sterilized and sown on MS medium containing 25 mg·L^−1^ Hyb, followed by cultivation for 3 weeks. The seedlings were then transplanted into 12 cm diameter pots containing a substrate mixture of peat soil, vermiculite, and perlite (3:1:1, *v*/*v*/*v*). After an additional 2 weeks of growth, plants with uniform development were selected for salt-stress treatment. During the treatment period, 100 mL of 300 mM NaCl solution was slowly applied to the soil every other morning, whereas the control plants received an equal volume of distilled water. Plant phenotype was recorded at 0, 7, 14, and 21 d of treatment, and biomass and related physiological parameters were measured at 21 d.

### 4.7. Measurement of Physiological Parameters and Related Gene Expression Levels

To systematically evaluate the effects of *RcnsLTPC* overexpression and ZnO-NPs priming on tobacco salt tolerance, physiological parameters were measured in both seedlings and adult plants after salt-stress treatment. Seedling root systems were scanned at high resolution using a root scanner (Expression 11000XL; Epson, Suwa, Japan), and total root length, branching number, average root diameter, root surface area, and root volume were quantified from the scanned images. In adult plants, the fresh weight (FW) and dry weight (DW) of leaves and roots were determined. The root/shoot ratio (R/S), water content (WC), dry matter content (DM), saturated fresh weight (SFW), relative water content (RWC), and water saturation deficit (WSD) were calculated accordingly.

Membrane lipid peroxidation and membrane integrity were evaluated by measuring malondialdehyde (MDA) content and relative electrolyte conductivity (REC), respectively. MDA content and antioxidant enzyme activities were determined as described previously [19]. REC was measured using the electrolyte leakage method as described by Lutts et al. (1996) [50], with REC (%) = (R1 − R3)/(R2 − R4) × 100, where R1 and R2 represent the initial and boiled conductivities of the sample, and R3 and R4 represent those of the deionized water blank.

ROS levels were assessed by measuring H_2_O_2_ and O_2_^−^ concentrations using commercial kits (Cat. No. G0112F and G0116F, respectively; Comin Biotechnology, Suzhou, China) according to the manufacturer’s instructions. ROS accumulation was further visualized by DAB and NBT histochemical staining as described by Thordal-Christensen et al. (1997) [51] and Doke (1983) [52], respectively. Leaf discs were vacuum-infiltrated for 30 min in either 1 mg·mL^−1^ DAB solution (0.1 M sodium phosphate buffer, pH 3.8) or 1 mg·mL^−1^ NBT solution (10 mM phosphate buffer, pH 7.8), incubated at 25 °C in the dark for 5–6 h, and then destained by boiling in 95% ethanol. Images were captured under a stereomicroscope (DVM6 S; Leica, Wetzlar, Germany).

Na^+^ and K^+^ contents in leaves and roots were determined by microwave plasma atomic emission spectrometry (4210 MP-AES; Agilent Technologies, Santa Clara, CA, USA). Dried tissue samples were digested using HNO_3_/HClO_4_ (4:1, *v*/*v*), and ion concentrations were quantified as described previously [53]. Expression levels of stress-related genes (*NtSOD*, *NtPOD*, *NtCAT*, *NtNHX1*, and *NtHKT1*) were analyzed by qRT-PCR using NtActin as the reference gene.

### 4.8. Statistical Analysis

Statistical analyses were performed using SPSS Statistics 27.0 (IBM Corp., Armonk, NY, USA). Data are presented as the mean ± standard deviation (SD). Statistical significance was determined using Tukey’s multiple comparison test at *p* < 0.05. All graphs were generated using Origin 2023 (OriginLab Corp., Northampton, MA, USA).

## 5. Conclusions

In this study, a new member of the nsLTP family, RcnsLTPC, was cloned and characterized from germinating castor seeds. RcnsLTPC was confirmed to encode a plasma membrane-localized basic secretory protein, and its promoter region was found to be enriched with multiple cis-elements responsive to various abiotic stresses. Functional validation demonstrated that heterologous overexpression of *RcnsLTPC* significantly enhanced salt tolerance in both yeast and transgenic tobacco (Figure 9). Its mechanism involves three synergistic pathways: activation of the antioxidant enzyme defense system to scavenge ROS, upregulation of ion transport-related genes to maintain Na^+^/K^+^ homeostasis, and promotion of root morphogenesis under salt stress. Furthermore, a significant synergistic effect was observed between ZnO-NPs priming and *RcnsLTPC* overexpression, with the combined treatment outperforming individual treatments across multiple key salt tolerance indicators. Collectively, these results indicate that *RcnsLTPC* functions as a positive regulator of plant salt tolerance through multi-pathway coordination, providing a potential target gene for molecular breeding of salt-tolerant castor and offering new experimental evidence for the functional diversification of the nsLTP family.

## Figures and Tables

**Table 1:** Physicochemical properties of the RcnsLTPC protein.

Parameter	Value
Total number of amino acids	175
Molecular weight (Da)	19.15 kDa
Theoretical pI	9.48
Instability index	26.16
Aliphatic index	79.20
Grand average of hydropathicity (GRAVY)	−0.217

**Table 2:** Final germination rate (%) of tobacco seeds under different NaCl concentrations.

Treatment	NaCl (mM)	WT	EV	OE-11	OE-12
CK	0	98.61 ± 1.96 a	98.61 ± 1.96 a	100.00 ± 0.00 a	98.61 ± 1.96 a
	50	81.94 ± 11.95 a	88.89 ± 5.20 a	95.83 ± 3.40 a	97.22 ± 3.93 a
	100	22.22 ± 1.96 b	18.06 ± 1.96 b	38.89 ± 3.93 a	37.50 ± 3.40 a
	150	6.94 ± 1.96 b	1.39 ± 1.96 b	26.39 ± 5.20 a	29.17 ± 3.40 a
ZnO-NPs	0	100.00 ± 0.00 a	100.00 ± 0.00 a	100.00 ± 0.00 a	100.00 ± 0.00 a
	50	93.06 ± 1.96 a	95.83 ± 3.40 a	94.44 ± 5.20 a	98.61 ± 1.96 a
	100	29.17 ± 3.40 b	25.00 ± 3.40 b	54.17 ± 3.40 a	55.56 ± 3.93 a
	150	9.72 ± 1.96 b	5.56 ± 1.96 b	30.56 ± 3.93 a	31.94 ± 1.96 a

Note: Data are presented as mean values. Different lowercase letters indicate significant differences among treatments at *p* < 0.05 level by Tukey’s test.

**Table 3:** Salt injury index (%) of tobacco seeds.

Treatment	NaCl (mM)	WT	EV	OE-11	OE-12
CK	50	16.90 ± 7.59 a	9.86 ± 3.65 ab	4.17 ± 2.41 ab	1.41 ± 1.45 b
	100	77.47 ± 1.27 a	81.69 ± 1.29 a	61.11 ± 2.78 b	61.97 ± 2.94 b
	150	92.96 ± 1.45 a	98.59 ± 1.39 a	73.61 ± 3.67 b	70.42 ± 2.83 b
ZnO-NPs	50	6.94 ± 1.39 a	4.17 ± 2.41 a	5.56 ± 3.67 a	1.39 ± 1.39 a
	100	70.83 ± 2.41 a	75.00 ± 2.41 a	45.83 ± 2.41 b	44.44 ± 2.78 b
	150	90.28 ± 1.39 a	94.44 ± 1.39 a	69.44 ± 2.78 b	68.06 ± 1.39 b

Note: Data are presented as mean values. Different lowercase letters indicate significant differences among treatments at *p* < 0.05 level by Tukey’s test.

**Table 4:** Effects of *RcnsLTPC* overexpression and ZnO-NPs priming on Na^+^ and K^+^ content in tobacco.

Group	Shoot			Root		
	Na^+^ (mg·kg^−1^)	K^+^ (mg·kg^−1^)	Na^+^/K^+^	Na^+^ (mg·kg^−1^)	K^+^ (mg·kg^−1^)	Na^+^/K^+^
CK, WT	103.00 ± 6.43 a	25.12 ± 3.61 b	4.23 ± 0.43 ab	89.33 ± 5.93 a	17.67 ± 1.45 b	5.07 ± 0.09 a
CK, EV	100.33 ± 7.88 a	21.33 ± 1.45 b	4.73 ± 0.41 a	90.32 ± 4.16 a	19.33 ± 1.13 b	4.68 ± 0.25 a
CK, OE-11	41.33 ± 3.53 c	35.32 ± 2.89 a	1.19 ± 0.09 c	50.16 ± 2.89 d	34.15 ± 1.20 a	1.45 ± 0.04 cd
CK, OE-12	41.86 ± 1.73 c	35.33 ± 1.45 a	1.19 ± 0.05 c	52.42 ± 2.31 cd	33.33 ± 1.76 a	1.56 ± 0.02 c
ZnO-NPs, WT	74.12 ± 4.93 b	23.33 ± 2.18 b	3.27 ± 0.54 b	64.00 ± 5.51 bc	22.67 ± 1.86 b	2.93 ± 0.43 b
ZnO-NPs, EV	75.32 ± 4.04 b	22.67 ± 2.60 b	3.37 ± 0.31 b	66.67 ± 4.81 b	22.19 ± 1.41 b	2.99 ± 0.14 b
ZnO-NPs, OE-11	31.00 ± 2.65 c	39.85 ± 2.60 a	0.78 ± 0.02 c	29.67 ± 2.60 e	35.33 ± 1.45 a	0.85 ± 0.10 d
ZnO-NPs, OE-12	32.33 ± 1.76 c	40.76 ± 1.53 a	0.79 ± 0.03 c	28.33 ± 4.06 e	34.67 ± 2.60 a	0.84 ± 0.15 d

Note: Data are presented as mean values. Different lowercase letters indicate significant differences among treatments at *p* < 0.05 level by Tukey’s test.

## Data Availability

The original contributions presented in this study are included in the article/Supplementary Material. Further inquiries can be directed to the corresponding authors.

## Supplementary Materials

The following supporting information can be downloaded at: https://www.mdpi.com/article/10.3390/plants15121827/s1. Figure S1. Construction of plant expression vectors. Schematic diagrams of the binary vectors pVS1 (A), pBI121 (B), and the yeast shuttle vector pYES2 (C) used in this study. Figure S2. Bioinformatic analysis of the RcnsLTPC protein. Hydropathy profile generated using the Kyte–Doolittle algorithm (A), illustrating the relationship between amino acid position and hydropathy score (B). Bar chart showing the frequency distribution of the 20 standard amino acids in the protein sequence. Figure S3. Molecular validation of recombinant vector construction. Electrophoretic analysis of PCR-amplified products of the RcnsLTPC gene coding region (A), where lane M is the DL2000 DNA marker (band sizes in bp) and lanes 1–3 show the target fragments; colony PCR validation of plant expression vector construction (B), with lanes 1–2 representing positive clones and lanes 3–4 showing empty vector controls; colony PCR validation of yeast expression vector construction (C), where lanes 1–4 display the target fragments. Figure S4. Effects of RcnsLTPC overexpression and ZnO-NP priming on antioxidant enzyme activities in tobacco under salt stress. Activities of superoxide dismutase (SOD) (A), peroxidase (POD) (B), catalase (CAT) (C), and ascorbate peroxidase (APX) (D) in leaves and roots, without (CK) or with ZnO-NP priming. Data are presented as mean ± SD (*n* = 3). Different lowercase letters indicate significant differences (*p* < 0.05, Tukey’s test). In the bar charts, the upper and lower parts represent data from shoots and broots tissues, respectively. Figure S5. Expression profiles of stress-related genes in WT and OE tobacco under 300 mM NaCl treatment. Data are presented as mean ± SE (*n* = 3). Different lowercase letters indicate significant differences among treatments (*p* < 0.05, Tukey’s test). Table S1. List of primer sequences. Table S2. Online analysis software name and website. Table S3. Summary of key bioinformatic prediction results for the RcnsLTPC protein. Table S4. Effects of *RcnsLTPC* overexpression and ZnO-NPs priming on tobacco salt tolerance (biomass and water status).
