# Supplementary material for: Castor RcnsLTPC Confers Salt Tolerance in Yeast and Tobacco with Synergistic Enhancement by ZnO-NPs Priming
Source: Plants (Basel). 2026 Jun 12;15(12):1827. doi: 10.3390/plants15121827 (PMC13306296; doi:10.3390/plants15121827)
Supplement: Supplementary file 1 [file plants-15-01827-s001.zip › Fig. S.pdf]

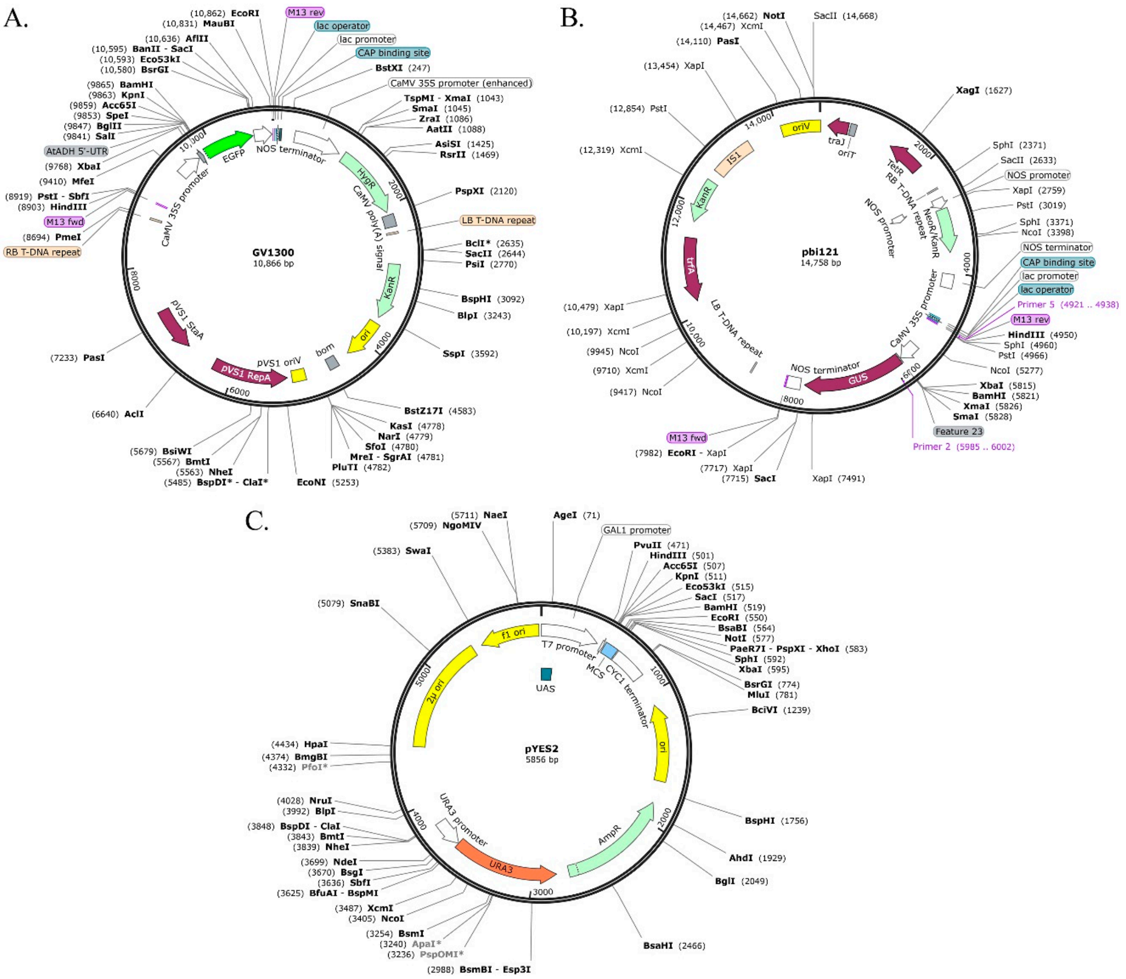

Figure S1 Construction of plant expression vectors. Schematic diagrams of the binary vectors pVS1 (A), pBI121 (B), and the yeast shuttle vector pYES2 (C) used in this study.

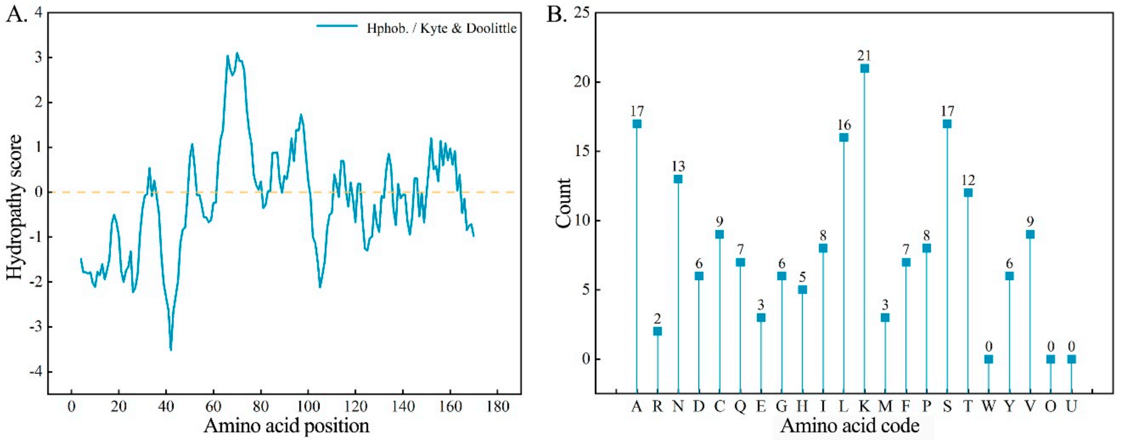

Figure S2 Bioinformatic analysis of the RcnsLTPC protein. Hydropathy profile generated using the Kyte–Doolittle algorithm (A), illustrating the relationship between amino acid position and hydropathy score (B). Bar chart showing the frequency distribution of the 20 standard amino acids in

the protein sequence.

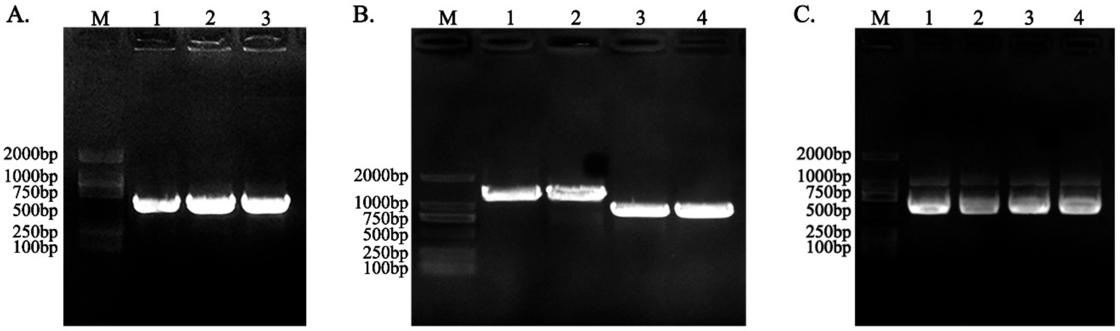

Figure S3 Molecular validation of recombinant vector construction. Electrophoretic analysis of PCR-amplified products of the *RcnsLTPC* gene coding region (A), where lane M is the DL2000 DNA marker (band sizes in bp) and lanes 1–3 show the target fragments; colony PCR validation of plant expression vector construction (B), with lanes 1–2 representing positive clones and lanes 3–4 showing empty vector controls; colony PCR validation of yeast expression vector construction (C), where lanes 1–4 display the target fragments.

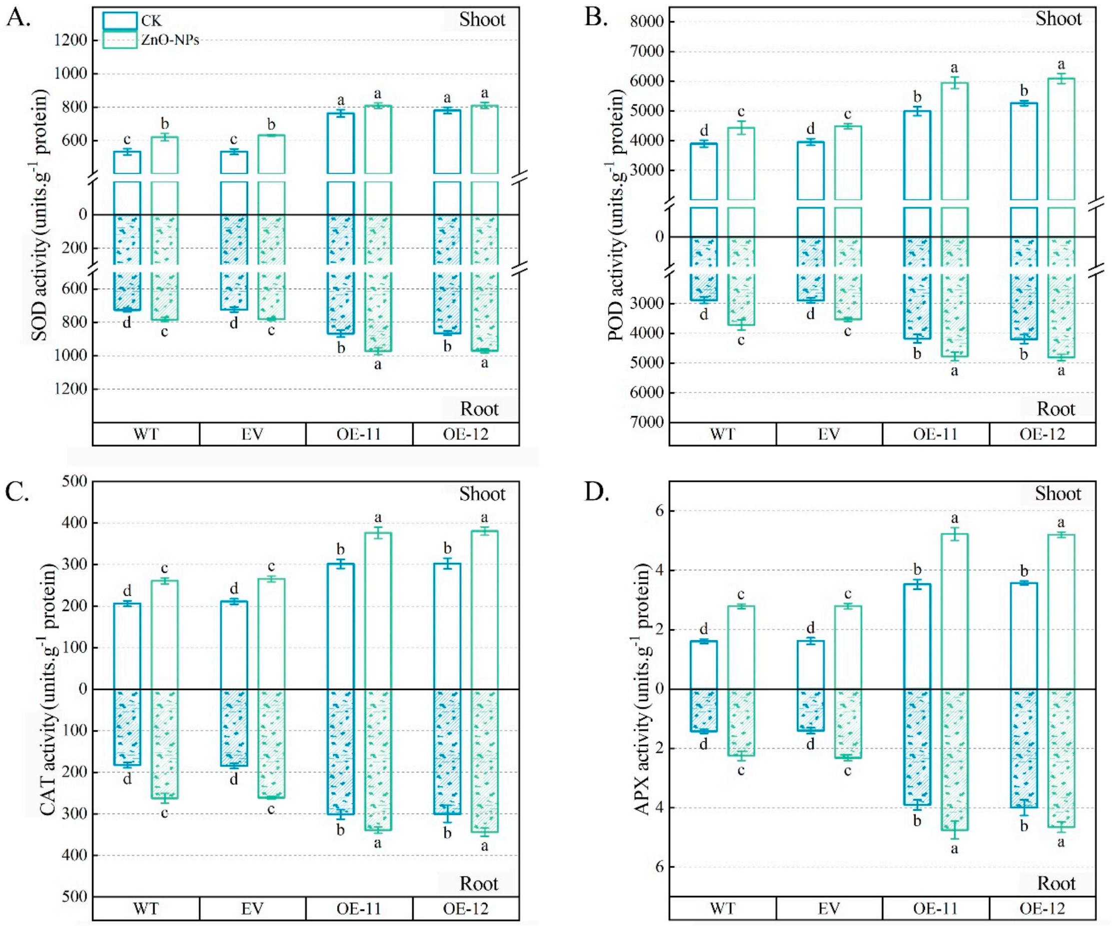

Figure S4 Effects of *RcnsLTPC* overexpression and ZnO-NP priming on antioxidant enzyme activities in tobacco under salt stress. Activities of superoxide dismutase (SOD) (A), peroxidase (POD) (B),

catalase (CAT) (C), and ascorbate peroxidase (APX) (D) in leaves and roots, without (CK) or with ZnO-NP priming. Data are presented as mean ± SD (n = 3). Different lowercase letters indicate significant differences (*P* < 0.05, Tukey's test). In the bar charts, the upper and lower parts represent data from shoots and broots tissues, respectively.

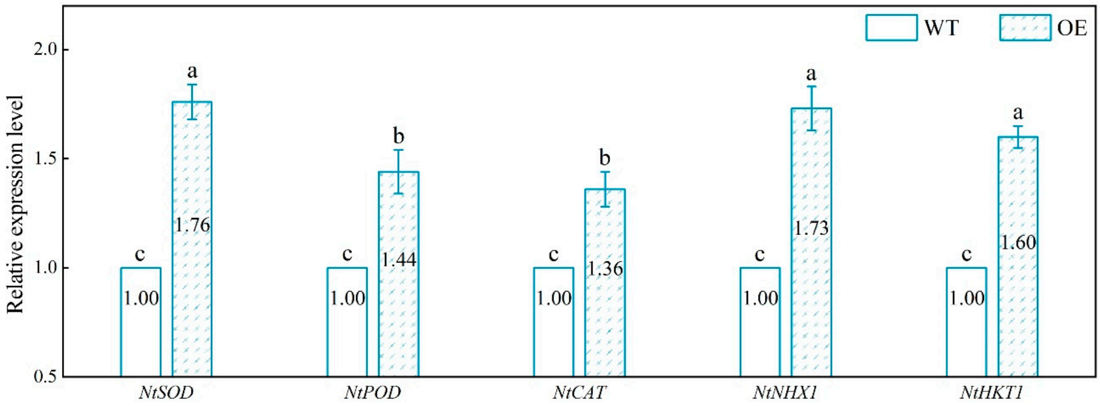

Figure S5 Expression profiles of stress-related genes in WT and OE tobacco under 300 mM NaCl treatment. Data are presented as mean ± SE (n = 3). Different lowercase letters indicate significant differences among treatments (*P* < 0.05, Tukey's test).
