# Supplementary material for: Castor RcnsLTPC Confers Salt Tolerance in Yeast and Tobacco with Synergistic Enhancement by ZnO-NPs Priming
Source: Plants (Basel). 2026 Jun 12;15(12):1827. doi: 10.3390/plants15121827 (PMC13306296; doi:10.3390/plants15121827)
Supplement: Supplementary file 1 [file plants-15-01827-s001.zip › Table S.pdf]

Table S1 List of primer sequences

Primer name	Forward primer (5′–3′)	Reverse primer (5′–3′)
RcnsLTPC	GCAACTTATCCATGCAGCATCAGTATAG	TGGCCTAATGAATCGTTTCGCAGTTGGT
*RcnsLTPC-Sal*I	GAGTCGACATGCAGCATCAGTATAC	
*RcnsLTPC-Bam*HI	AGGATCCGATCCTATGAATCGTTTCGCAGT	
pro-*RcnsLTPC*	ACGAGCAGAGTTCGAACTTAGTTCAT	GGATAAGTTGCATTGGGACTGAAACC
pYES2-*RcnsLTPC*	CTTGGTACCGAGCTCGGATCCATGCAGCATCA GTATAGCAA AATTAA	TACATGATGCGGCCCTCTAGACTAATGAATCG TTTCGCAGT TGG

Table S2 Online analysis software name and website

Online tools	Features	Website
CD Search	Conserved domain analysis	https://www.ncbi.nlm.nih.gov/Structure/cdd/wrpsb.cgi
Prot Param	Physicochemical property analysis	https://web.expasy.org/protparam/
Prot Scale	Hydrophilicity analysis	https://web.expasy.org/protscale/
TMHMM2.0	Transmembrane structure analysis	https://services.healthtech.dtu.dk/service.php?TMHMM-2.0
Signal P6.0	Signal peptide analysis	https://services.healthtech.dtu.dk/service.php?SignalP-6.0
NetNGlyc-1.0	Glycosylation sites	https://services.healthtech.dtu.dk/service.php?NetNGlyc-1.0
MusiteDeep	Phosphorylation sites<br>Acetylation sites<br>Ubiquitination sites<br>SUMOylation sites	https://www.musite.net/
SOPMA	Protein secondary structure prediction	https://npsa-prabi.ibcp.fr/
SWISS MODEL	Protein tertiary structure prediction	https://www.swissmodel.expasy.org/
DeepLoc 2.0 and TargetP 2.0	Subcellular localization prediction	https://services.healthtech.dtu.dk/service.php?DeepLoc2.0和h ttps://services.healthtech.dtu.dk/service.php?TargetP-2.0
PlantCARE	Promoter analysis	http://bioinformatics.psb.ugent.be/webtools/plantcare/html/

Table S3 Summary of key bioinformatic prediction results for the RcnsLTPC protein

Prediction Item	Main Result	Key Parameter / Probability
Signal peptide	No (SignalP 6.0)	Other probability: 0.9996
	Yes (DeepLoc 2.0)	Predicted signals: Signal peptide
Transmembrane domain	Yes (1 domain)	Position: Residues 63–85<br>Exp number: 19.59
Subcellular localization	Extracellular	Probability: 0.6211
	Endoplasmic reticulum	Probability: 0.3774
	Lysosome/Vacuole	Probability: 0.3282
	Nucleus	Probability: 0.1624
	Cell membrane	Probability: 0.1308
Secretory pathway	Secretory pathway	Reliability Class (RC): 1

Table S4 Effects of *RcnsLTPC* overexpression and ZnO-NPs priming on tobacco salt tolerance (biomass and water status)

Group	Root-shoot ratio	Leaf water content	Root water content	Leaf dry matter	Root dry matter	Relative water	Water saturation

				content	content	content (%)	deficit (%)
CK,WT	0.0288 ± 0.0026e	0.8827 ± 0.0005c	0.9685 ± 0.0027a	0.1173 ± 0.0005a	0.0315 ± 0.0027e	63.35 ± 1.17c	36.65 ± 1.17a
CK,EV	0.0489 ± 0.0023c	0.8893 ± 0.0011c	0.9621 ± 0.0012bc	0.1107 ± 0.0011a	0.0379 ± 0.0012cd	64.38 ± 0.53c	35.62 ± 0.53a
CK,OE-11	0.0670 ± 0.0019a	0.8996 ± 0.0004b	0.9591 ± 0.0011cd	0.1004 ± 0.0004b	0.0409 ± 0.0011bc	71.50 ± 1.21ab	28.50 ± 1.21bc
CK,OE-12	0.0695 ± 0.0033a	0.8966 ± 0.0025b	0.9567 ± 0.0012d	0.1034 ± 0.0025b	0.0433 ± 0.0012b	70.13 ± 1.23ab	29.87 ± 1.23bc
ZnO-NPs,WT	0.0631 ± 0.0021ab	0.9040 ± 0.0012b	0.9606 ± 0.0022bcd	0.0960 ± 0.0012b	0.0394 ± 0.0022bcd	68.70 ± 1.06b	31.30 ± 1.06b
ZnO-NPs,EV	0.0629 ± 0.0024ab	0.9013 ± 0.0044b	0.9505 ± 0.0015e	0.0987 ± 0.0044b	0.0495 ± 0.0015a	68.12 ± 1.01b	31.88 ± 1.01b
ZnO-NPs,OE-11	0.0593 ± 0.0009b	0.9229 ± 0.0008a	0.9457 ± 0.0017e	0.0771 ± 0.0008c	0.0543 ± 0.0017a	72.80 ± 0.71a	27.20 ± 0. 71c
ZnO-NPs,OE-12	0.0422 ± 0.0005d	0.9020 ± 0.0034b	0.9647 ± 0.001ab	0.0980 ± 0.0034b	0.0353 ± 0.0010de	72.80 ± 1.97a	27.20 ± 1.97c

Note: Data are presented as mean values. Different lowercase letters indicate significant differences among treatments at $P < 0.05$ level by Tukey's test.
