# Supplementary figures and images for: Castor RcnsLTPC Confers Salt Tolerance in Yeast and Tobacco with Synergistic Enhancement by ZnO-NPs Priming
Source: Plants (Basel). 2026 Jun 12;15(12):1827. doi: 10.3390/plants15121827 (PMC13306296; doi:10.3390/plants15121827)

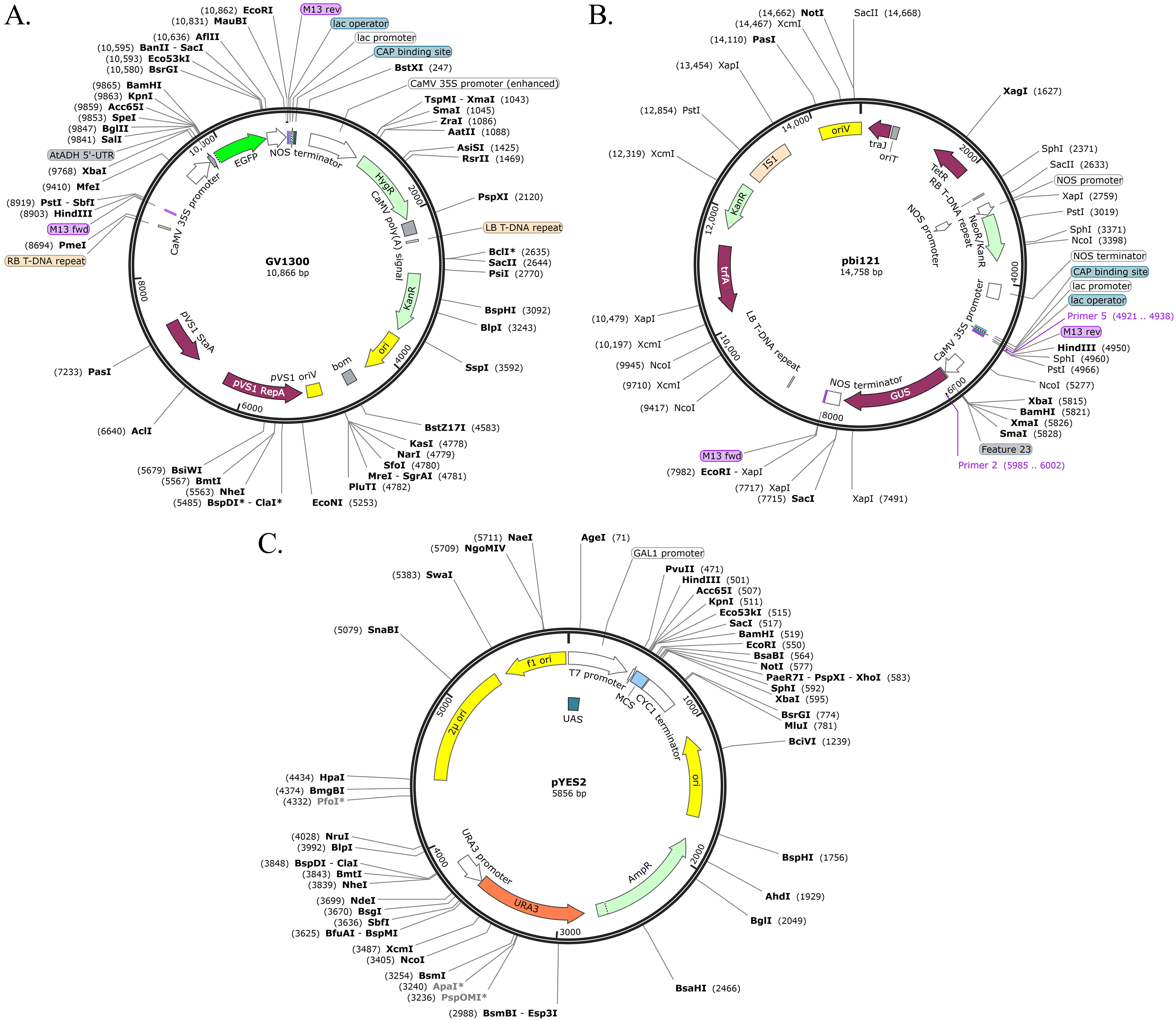

Supplement: Supplementary file 1 [file plants-15-01827-s001.zip › Fig.S/Fig.S1.jpg]

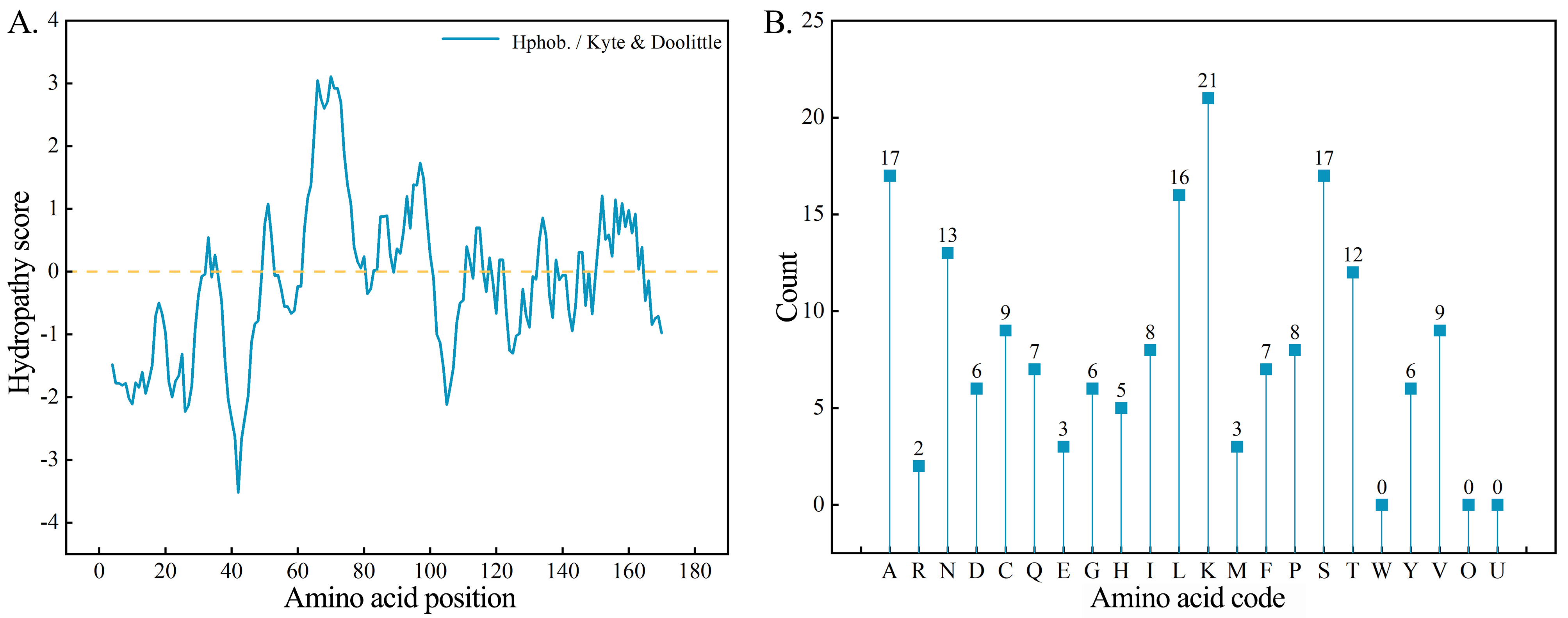

Supplement: Supplementary file 1 [file plants-15-01827-s001.zip › Fig.S/Fig.S2.jpg]

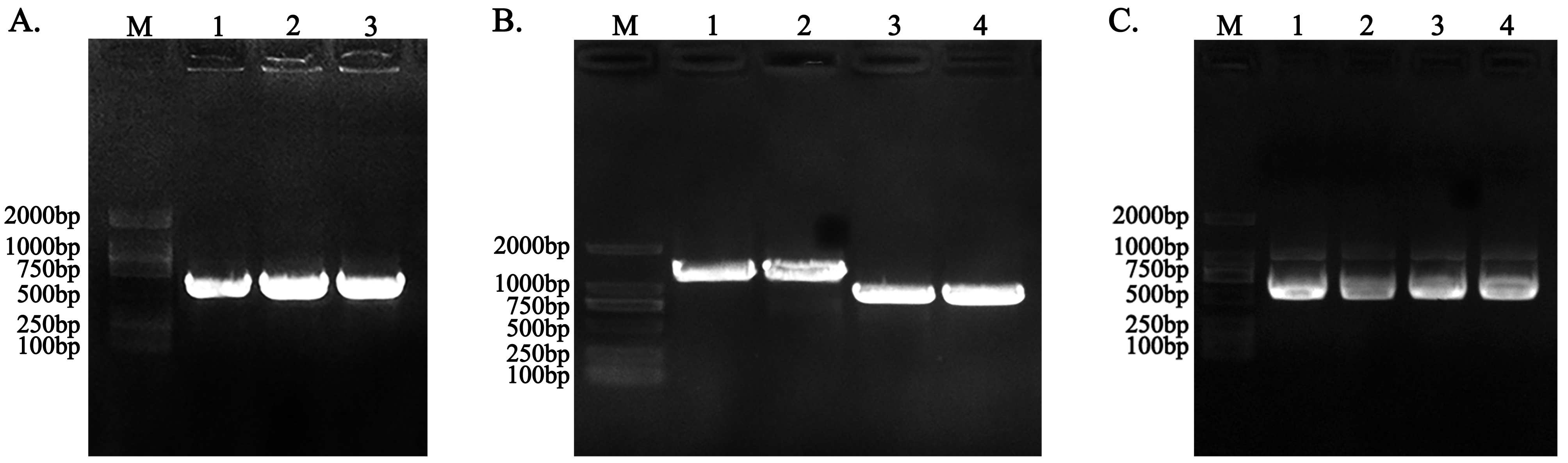

Supplement: Supplementary file 1 [file plants-15-01827-s001.zip › Fig.S/Fig.S3.jpg]

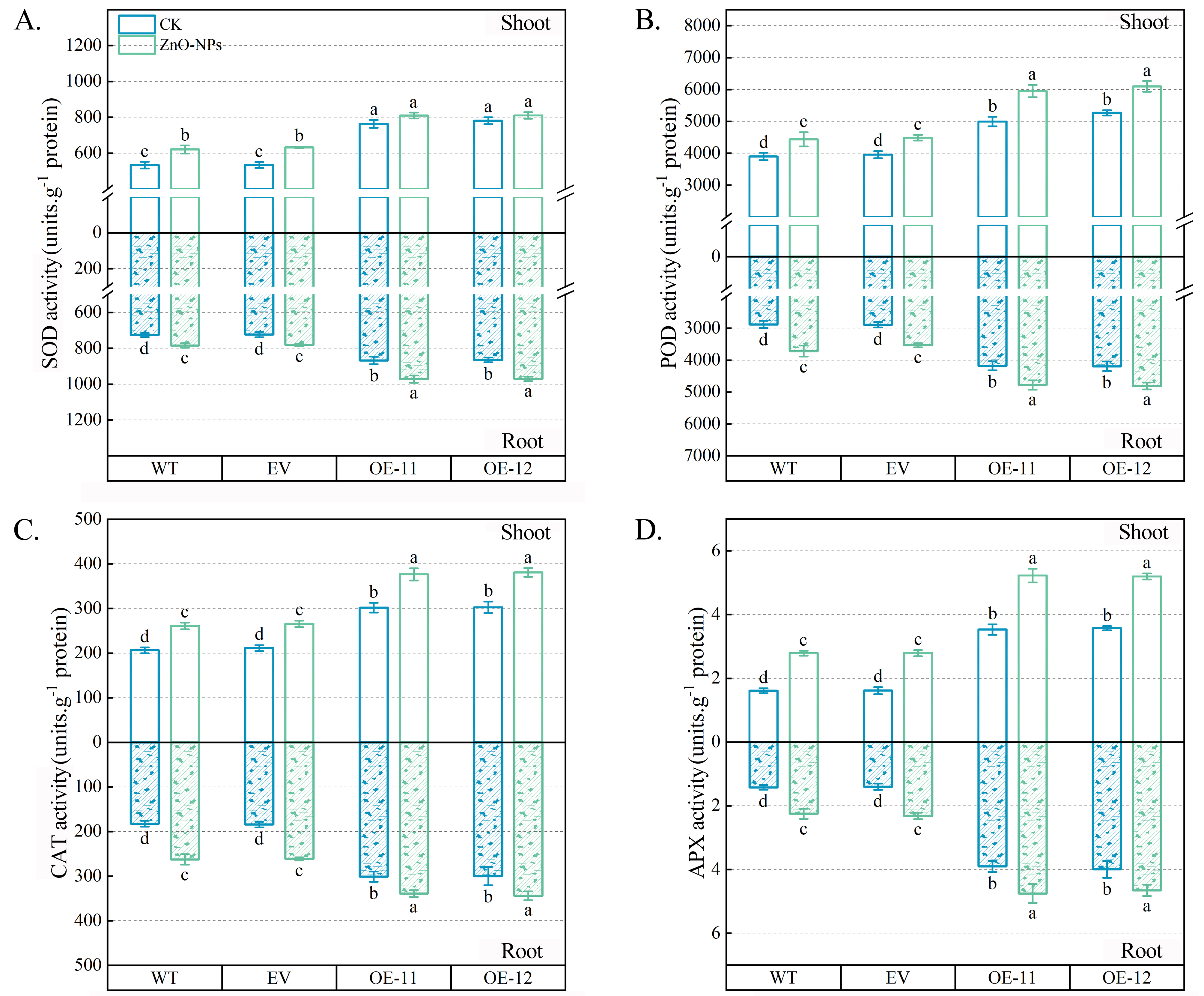

Supplement: Supplementary file 1 [file plants-15-01827-s001.zip › Fig.S/Fig.S4.jpg]

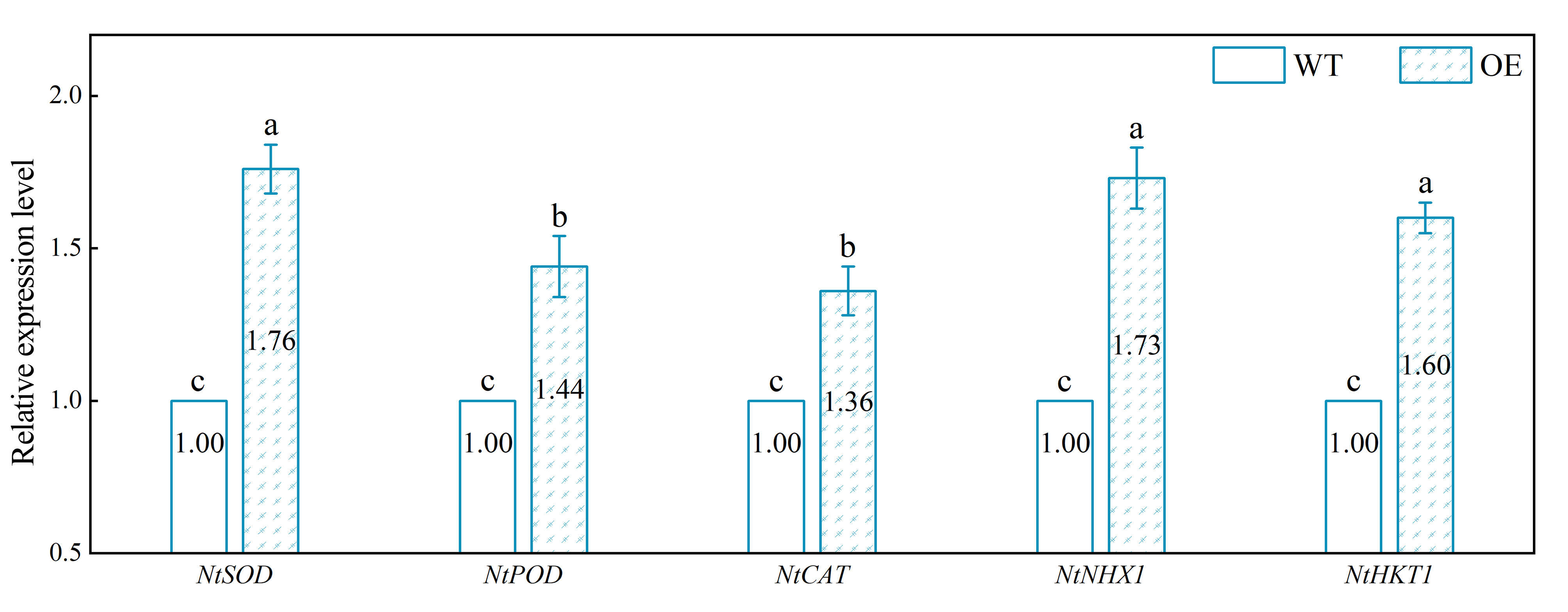

Supplement: Supplementary file 1 [file plants-15-01827-s001.zip › Fig.S/Fig.S5.jpg]
